# DeepITEH: a deep learning framework for identifying tissue-specific eRNAs from the human genome

**DOI:** 10.1093/bioinformatics/btad375

**Published:** 2023-06-09

**Authors:** Tianjiao Zhang, Liangyu Li, Hailong Sun, Guohua Wang

**Affiliations:** College of Computer and Control Engineering, Northeast Forestry University, Harbin 150040, China; College of Computer and Control Engineering, Northeast Forestry University, Harbin 150040, China; College of Computer and Control Engineering, Northeast Forestry University, Harbin 150040, China; College of Computer and Control Engineering, Northeast Forestry University, Harbin 150040, China

## Abstract

**Motivation:**

Enhancers are vital cis-regulatory elements that regulate gene expression. Enhancer RNAs (eRNAs), a type of long noncoding RNAs, are transcribed from enhancer regions in the genome. The tissue-specific expression of eRNAs is crucial in the regulation of gene expression and cancer development. The methods that identify eRNAs based solely on genomic sequence data have high error rates because they do not account for tissue specificity. Specific histone modifications associated with eRNAs offer valuable information for their identification. However, identification of eRNAs using histone modification data requires the use of both RNA-seq and histone modification data. Unfortunately, many public datasets contain only one of these components, which impedes the accurate identification of eRNAs.

**Results:**

We introduce DeepITEH, a deep learning framework that leverages RNA-seq data and histone modification data from multiple samples of the same tissue to enhance the accuracy of identifying eRNAs. Specifically, deepITEH initially categorizes eRNAs into two classes, namely, regularly expressed eRNAs and accidental eRNAs, using histone modification data from multiple samples of the same tissue. Thereafter, it integrates both sequence and histone modification features to identify eRNAs in specific tissues. To evaluate the performance of DeepITEH, we compared it with four existing state-of-the-art enhancer prediction methods, SeqPose, iEnhancer-RD, LSTMAtt, and FRL, on four normal tissues and four cancer tissues. Remarkably, seven of these tissues demonstrated a substantially improved specific eRNA prediction performance with DeepITEH, when compared with other methods. Our findings suggest that DeepITEH can effectively predict potential eRNAs on the human genome, providing insights for studying the eRNA function in cancer.

**Availability and implementation:**

The source code and dataset of DeepITEH have been uploaded to https://github.com/lyli1013/DeepITEH.

## 1 Introduction

The regulation of gene expression is a complex process influenced by various factors. Among them, enhancers play a crucial role as cis-acting elements involved in the transcriptional regulation of genes, leading to the enhanced expression of target genes (Plank and Dean 2014). The direction-independent function of enhancers is often observed, and they typically act from a distance from their target genes (Heintzman and Ren 2009). Several cancers and diseases in humans have been linked to the abnormal expression of enhancers (Sakabe *et al.* 2012, Nasser *et al.* 2021). Recent investigations have revealed that validated enhancers also generate long noncoding RNAs (lncRNAs), commonly known as enhancer RNAs (eRNAs) (Han and Li 2022). Consequently, eRNAs are a group of enhancers with expression activity, which serve as critical regulatory elements of gene expression by enhancing the expression of their target genes (Pnueli *et al.* 2015). Furthermore, eRNAs exhibit strong tissue specificity, restricted to specific tissues, cells, and environmental conditions (Andersson *et al.* 2014, Sartorelli and Lauberth 2020). Aberrant expression of key eRNAs has been implicated in various diseases (Lee *et al.* 2020), including cancer, cardiovascular diseases, and metabolic diseases. Therefore, the precise identification of these eRNAs is critical to understanding their mechanisms of action in related diseases.

Computational approaches for enhancer identification commonly involve extracting sequence features from the genome and constructing classification models for enhancer identification through machine learning or deep learning techniques (Liu *et al.* 2016, Tahir *et al.* 2018, Le *et al.* 2019). For instance, iEnhancer-2L (Liu *et al.* 2016) is machine learning models applied to enhancer identification. In recent years, deep learning-based methods have become prevalent due to the rapid growth of multiple biological data. In 2021, Mu *et al.* (2021) utilized the attention mechanism in a bidirectional long short-term memory (Bi-LSTM) model for the enhancer detection problem. Yang *et al.* (2021) applied deep neural networks (DNNs) to construct an iEnhenger-RD calculation method for enhancer identification in 2021. Similarly, in 2022, Huang *et al.* (2022) proposed a deep learning approach consisting of a deep residual neural network, Bi-LSTM, and feed-forward attention for enhancer recognition. Furthermore, Wang *et al.* (2022) proposed a prediction model for enhancers based on 10 feature encodings and five machine learning algorithms in 2022. However, these approaches that rely solely on sequence features for enhancer identification ignore the fact that enhancers with transcriptional activity are often highly tissue-specific, and specifically expressed enhancers exhibit greater functionality in specific tissues (Han and Li 2022). Therefore, the accuracy of identification methods that rely solely on enhancer sequences to identify tissue-specific expression enhancers may be compromised. To address this limitation, we can combine multiple biological signals (Zhou *et al.* 2011, Buecker and Wysocka 2012) on the genome that are closely related to enhancers as features with sequence features to construct prediction models. This requires both RNA-seq data of the genome and histone modification data. However, currently available public datasets often contain only one of these components, resulting in a lack of sufficient data support for recognizing tissue-specific expression of enhancers.

In this study, we present a novel deep learning framework, named DeepITEH, for accurately identifying tissue-specific eRNAs from the human genome. DeepITEH integrates RNA-seq data and histone modification data from multiple samples within the same tissue to improve the accuracy of eRNA identification. Initially, DeepITEH classifies eRNAs into regularly expressed eRNAs (RE) and accidental eRNAs (AE) based on histone modification data. Then, it combines histone modification features with sequence features to identify tissue-specific eRNAs. DeepITEH is evaluated on four datasets of normal tissues and four datasets of cancer tissues separately. The study finds that DeepITEH outperforms existing enhancer prediction methods on seven independent test sets, namely, stomach, lung, liver, pancreas, liver cancer, lung adenocarcinoma, and prostate cancer tissues. This demonstrates that DeepITEH can accurately identify eRNA in specific tissues.

## 2 Materials and methods

### 2.1 Enhancer RNA and nonenhancer data

The eRNA data of four normal tissues, namely, stomach, lung, liver, and pancreas, are obtained from the Human eRNA Database (HeRA) (Zhang *et al.* 2021). In addition, the eRNA data of four cancer tissues are obtained from the eRic database (Zhang *et al.* 2019), specifically liver-hepatocellular-carcinoma (LIHC), lung-adenocarcinoma (LUAD), prostate-adenocarcinoma (PRAD), and pancreatic-adenocarcinoma (PAAD). The eRNA data from both databases are in the form of DNA sequences of their respective enhancers. The Liftover tool in UCSC is used to convert the eRNA data to the hg19 version. The nonenhancer (NE) dataset is obtained from previous studies (Liu *et al.* 2016, Basith *et al.* 2021). For each tissue, NE data of the same number as eRNAs are randomly selected and expanded to sequences of equal length as eRNAs. The dataset is then split into training and independent test sets with a proportion of 4:1 for each tissue type. The sample size of each dataset is shown in Supplementary Table S1.

### 2.2 Histone modifications data

A previous study has suggested that certain genomic biosignals, including H3K4me1, H3K4me3, H3K9me3, and H3K27ac, are strongly correlated with eRNA (Heintzman *et al.* 2009, Creyghton *et al.* 2010, Pekowska *et al.* 2011, Wang *et al.* 2011). Specifically, these four biosignals tend to exhibit enrichment in active enhancer regions, and attract specific transcription factors and auxiliary protein complexes to regulate their own expression as well as that of genes. In contrast, H3K36me3 exhibits a negative correlation with eRNA (Koch *et al.* 2011, Bonn *et al.* 2012, Li *et al.* 2016), where the elevation of H3K36me3 levels may suppress eRNA production, subsequently affecting enhancer activation and the expression of downstream target genes. Given these observations, we selected these five histone modifications as eRNA recognition features. We download ChIP-seq data for the five selected histone modifications from the ENCODE (Luo *et al.* 2020) database. Supplementary Tables S2–S9 provide information about the histone modification data for the different samples of stomach, lung, liver, pancreas, LIHC, LUAD, PRAD, and PAAD tissues.

### 2.3 Framework of DeepITEH

**Figure 1:**
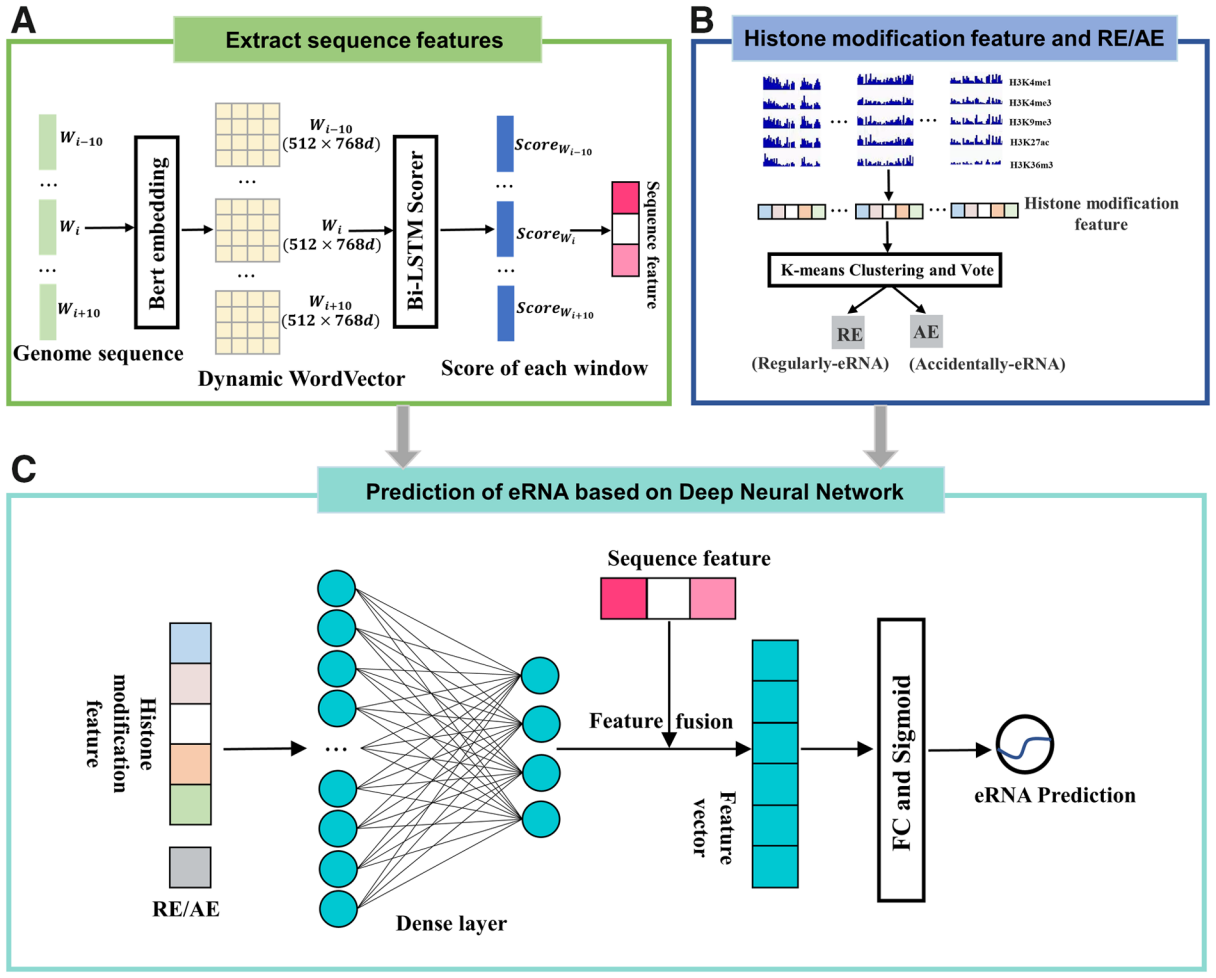
Framework of DeepITEH.

In order to identify tissue-specific eRNAs in the human genome, we employ a DNN with multiple biological features as input to quantify the probability of a sequence being an eRNA, as depicted in Fig. 1. Initially, deep learning Bert and Bi-LSTM techniques are employed to extract potential patterns of eRNA sequence information, i.e. sequence features of eRNAs (Fig. 1A). Subsequently, we cluster eRNAs using *k*-means based on the histone modification features of various samples in the same tissue, and classify eRNAs as either RE or AE via voting (Fig. 1B). Finally, the histone modification features and sequence features are combined and entered into a DNN to predict tissue-specific eRNAs (Fig. 1C).

#### 2.3.1 Determine the location of sequence features

**Figure 2:**
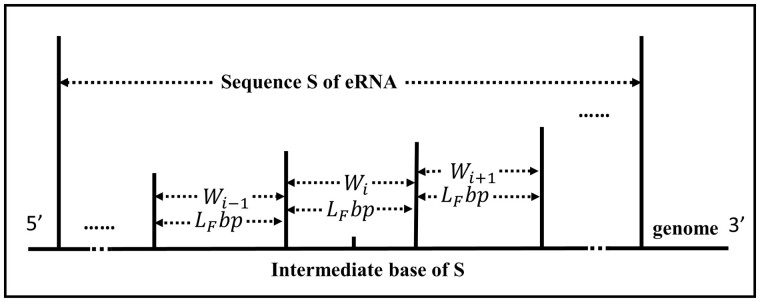
Delineation of the eRNA window for extracting sequence features.

**Figure 3:**
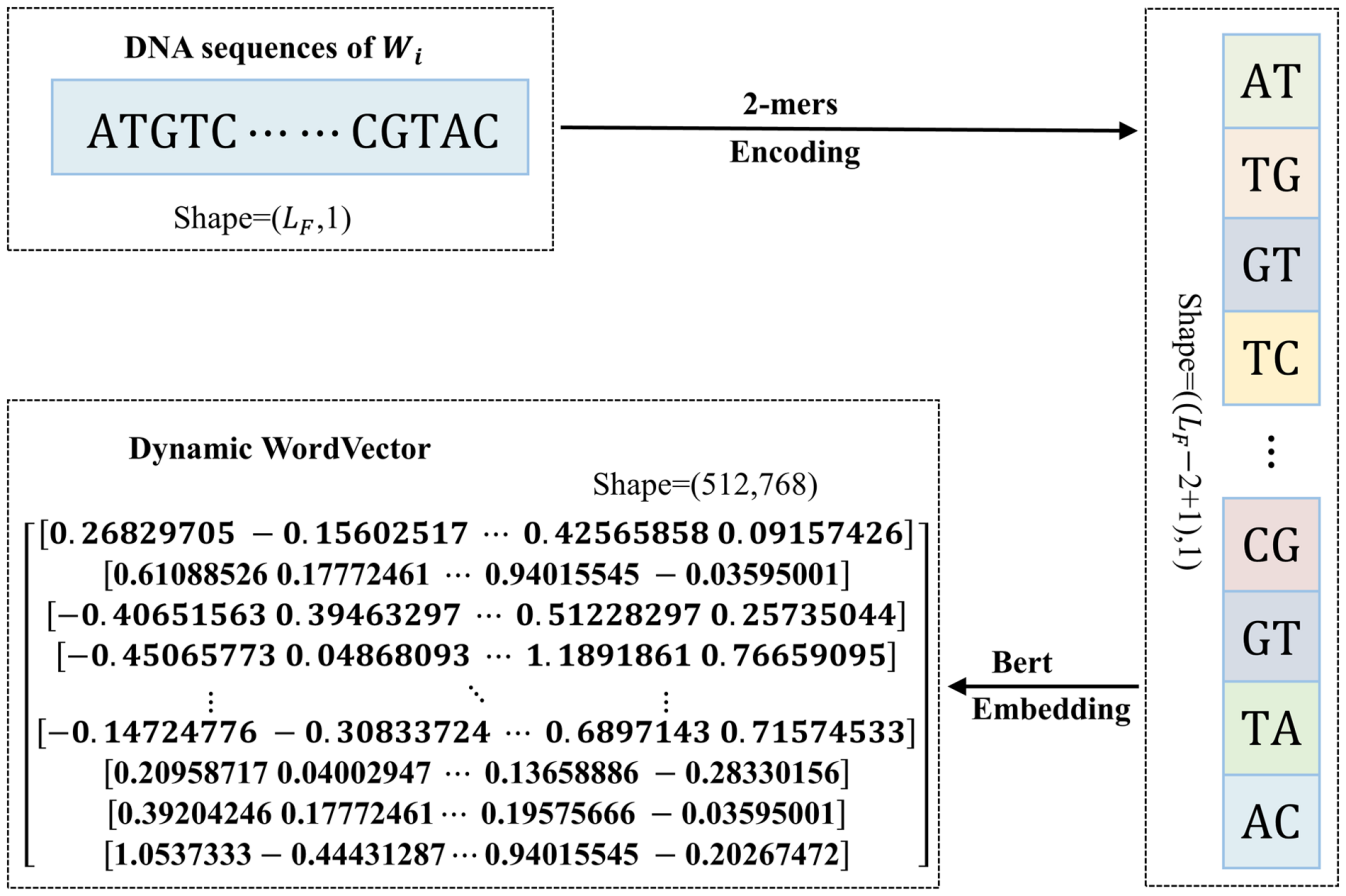
Bert Embedding.

In the HeRA and eRic databases, eRNA is defined as the ±3kb region of the original enhancer intermediate base, resulting in a sequence length of S=6001bp. Due to the excessive length and noise of the eRNA sequence, windowing is employed to segment the eRNA, followed by the selection of the most informative region that best represents the eRNA sequence. Subsequently, the quantification of DNA sequence features on eRNA/NE sequences (positive/negative set of examples) is presented as follows:

1. Determine the size of the division window. To determine the size of the division window, we rely on the original enhancer annotations of eRNAs obtained from the FANTOM (Lizio *et al.* 2019) (which uses the CAGE technique to define enhancers), ENCODE, and Roadmap Epigenomics Project (Chadwick 2012) (which use histone modification data to define enhancers). Specifically, the mean length of enhancers in the FANTOM dataset, denoted as $L_{F}$, is utilized as the window size to divide eRNAs.
2. The windowing procedure for eRNA sequences is as follows. In a similar manner to the definition of eRNA as the ±3kb region of enhancer intermediate bases in the HeRA and eRic databases, the $\pm L_{F}/2bp$ region of eRNA intermediate bases is utilized as the first window $W_{i}$. Subsequently, $L_{F}bp$ is taken upstream and downstream in turn to obtain $W_{i-1}$ and $W_{i+1}$, respectively, until the complete division of eRNA, resulting in $S=\left\{\cdots ,W_{i-1},W_{i},W_{i+1},\cdots \right\}$, as depicted in Fig. 2.
3. The optimal window that best characterizes the eRNA sequence is selected using a deep learning Bert+Bi-LSTM model. The DNA sequence is considered as a sentence and natural language processing is used to encode each word in the sentence as a vector. Bidirectional Encoder Representations from Transformers (BERT) (Devlin *et al.* 2018) is a natural language pretraining model developed by the Google AI Research Institute, which generates dynamic and contextual word vectors that can fully capture information and alignment of both upstream and downstream regions of DNA sequences. Initially, *k*-mers encoding is performed for each region in $S=\left\{\cdots ,W_{i-1},W_{i},W_{i+1},\cdots \right\}$. Specifically, a sliding window size of k (where k∈N+) and a step size of 1 are set, which divides each region in S into $\left(L_{F}-k+1\right)$ words of length kb. Due to the similar frequency and statistical properties of dimer nucleotides across various tissue-specific DNA sequences, and the limited number of 16 possible combinations for 2-mer coding, a *k* value of 2 is selected to improve computational efficiency and prevent overfitting Next, the pretraining model Bert is applied to each word to generate a dynamic word vector. As an example, Fig. 3 shows the region $W_{i}$ in S.
  The Bi-LSTM (Siami-Namini *et al.* 2019) network is a variant of the long short-term memory (LSTM) (Hochreiter and Schmidhuber 1997) network, in which each input sequence is processed through a recurrent neural network twice, once in the forward direction and once in the reverse direction. The bidirectional structure provides a stronger memory capacity and is effective in handling long sequence data. Therefore, it is widely used in tasks such as sequence prediction and natural language processing (Mustaqeem *et al.* 2020, Cai *et al.* 2021). As there is a certain pattern of dependence between the upstream and downstream of DNA sequences, we construct the window scorer based on the Bi-LSTM. First, the window word vectors are inputted into a Bi-LSTM with 64 hidden layer nodes, and batch normalization and Dropout regularization are applied to enhance computational speed and prevent overfitting. Subsequently, the feature vector's dimensional transformation is performed using the same Bi-LSTM configuration, and the output is given to the Dropout layer. Finally, the fully connected layer and the activation function Sigmoid output the score of the window. The dynamic word vector of each window is individually fed into this scorer, and the window with the highest score is selected as the key region for extracting sequence features.
4. Extraction of sequence features. The sequence feature $S_{\mathrm{feature}}$ of an eRNA or NE is extracted from the optimal window position using a scorer constructed based on Bi-LSTM.

#### 2.3.2 Determine the location of histone modification features

**Figure 4:**
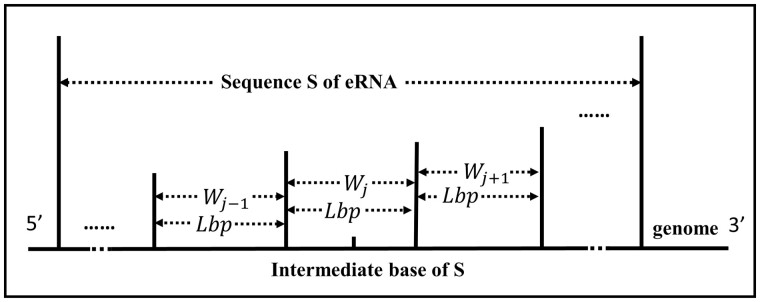
Delineation of the eRNA window for extracting histone modification features.

Due to its longer length, eRNA sequences often contain a significant amount of noise. Additionally, eRNAs defined in HeRA and eRic are derived not only from FANTOM, but also from histone modification data in ENCODE and Roadmap Epigenomics Project. Consequently, when dividing the window for extracting histone modification features of eRNAs, we must consider the enhancer definitions' lengths in the aforementioned three databases. The quantification of histone modification features on eRNA/NE sequences (positive/negative set of examples) is outlined as follows:

1. The first step in quantifying histone modification features on eRNA/NE sequences involves collecting data on the length of peaks for each type of histone modification in each tissue, as well as the average length of peaks $L_{\mathrm{peak}}$.
2. Determine the size of the division window. The determination of the window size for extracting histone modification features involves considering the average length $L_{F}$ of enhancers in the FANTOM database and the average length $L_{\mathrm{peak}}$ of histone modification data. If the value of $L_{\mathrm{peak}}$ is less than or equal to $L_{F}$, eRNA is divided using $L_{F}$ as the window size. Alternatively, if $L_{\mathrm{peak}}$ is greater than $L_{F}$, eRNA is divided using $L_{\mathrm{peak}}$ of this histone modification data as the window size. As a result of this window division rule, histone modification data for different samples of each tissue will lead to varying window division sizes.
3. Windowing for eRNA. The $\pm L/2bp(L\in \{L_{\mathrm{peak}},L_{F}\})$ region surrounding the intermediate bases of the eRNA is designated as the first window, $W_{j}$. Then windows are obtained by taking Lbp upstream and downstream in turn to create $W_{j-1}$ and $W_{j+1}$, respectively, until the eRNA is fully divided. This results in a set of windows, $S=\left\{\cdots ,W_{j-1},W_{j},W_{j+1},\cdots \right\}$, as illustrated in Fig. 4. Additionally, the distribution of each histone modification feature on every window of each eRNA, including the position, height, and width of the peaks, is statistically analyzed to validate the chosen window size.
4. Selection of the optimal window. The eRNAs of a given tissue are aligned and stacked, with the central base as the midpoint. Subsequently, all eRNAs are divided according to the window size L, determined by each type of histone modification data separately. The number of eRNAs containing the signal on each window is counted for each type of histone modification signal. Among the histone modifications closely related to eRNA, namely, H3K4me1, H3K4me3, H3K9me3, and H3K27ac, the window with the highest number of eRNAs is selected as the optimal window. In contrast, the window with the least number of eRNAs is chosen as the optimal window for H3K36me3, which is negatively correlated with eRNA.
5. Extraction of histone modification feature. Each tissue contains data on histone modifications for n(n∈N+) different samples. Using the selected optimal window, we extract the histone modification features of eRNAs from the histone modification data of different samples of each tissue individually. This yields n sets of histone modification features of eRNA for that tissue. Following the same methodology, we extract n sets of histone modification features for NEs in that tissue.

#### 2.3.3 Feature matrix

Our hypothesis posits that eRNAs expressed in a particular tissue at a particular time can be divided into two categories: RE and AE. We postulate that REs should exhibit consistent histone modification patterns across multiple histone modification experiments (ChIP-seq). To identify eRNAs with patterns similar to RE, we cluster and employ voting mechanisms to combine histone modification data from various samples.

1. The process for identifying REs from NE histone modification features and eRNA histone modification features extracted from the same sample of the same tissue involves several steps. First, the eRNA and NE histone modification features are spliced together to create a 5D feature matrix. This matrix is then used in the *k*-means machine learning algorithm to cluster the eRNAs and NEs into two separate classes, one for candidate REs and the other for candidate AEs or candidate NEs. As n sets of histone modification features can be obtained from n different samples of the same tissue, we use the sets of candidate REs obtained by clustering to select the true REs by applying a voting procedure. Specifically, if there are n(n is even) sets of candidate REs in the tissue and an eRNA is a candidate RE in all m sets where m belongs to the range [n/2, n], then the eRNA is considered the true RE of the tissue. If there are n(n is odd) sets of candidate REs in the tissue and an eRNA is a candidate RE in all m sets where m belongs to the range (n/2, n], then the eRNA is considered the true RE of the tissue.
2. To combine histone modification data from different samples, we add the RE, AE, and NE features as binary variables to the eRNA and NE histone modification features extracted from each sample. The RE feature value is set to 1, and the AE and NE feature values are set to 0, resulting in a 6D feature matrix.

#### 2.3.4 Deep neural network-based identification of enhancer RNA

**Figure 5:**
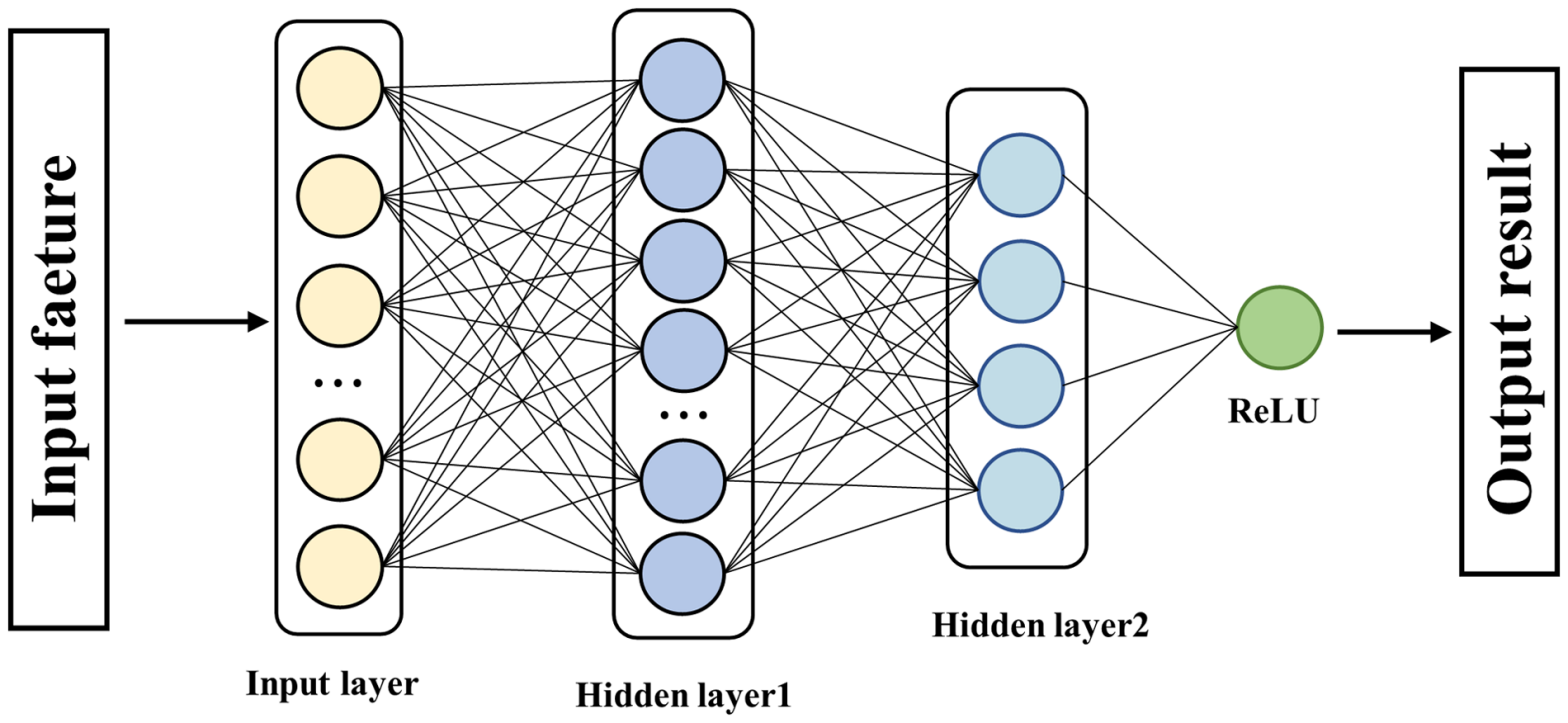
Deep neural network graph with two Dense layers.

For a given eRNA or NE sample $S_{i}$, we begin by constructing the 6D feature matrix using the previously described method, which serves as the input to a DNN. Specifically, we apply two Dense layers, each equipped with the ReLU activation function, to this input feature matrix, as illustrated in Fig. 5. To achieve this, we subject the input feature matrix $a^{l}=\left[a_{1}\;a_{2}\;a_{3}\;a_{4}\;a_{5}\;a_{6}\right]$ to a sequence of linear and activation operations using the weight coefficient matrix $W^{\left(l+1\right)}$ and the bias vector $b^{l+1}$, respectively, i.e. where the number of layers l=0,1,2, $W^{\left(l+1\right)}$ is the weight coefficient matrix of layer l+1, $b^{l+1}$ is the bias vector of layer l+1, and $a^{\left(l+1\right)}$ is the output matrix of layer l+1. In addition, to improve efficiency and prevent overfitting, we incorporate Dropout regularization.


(1) $$a^{\left(l+1\right)}=\mathrm{ReLU}\left(z^{\left(l+1\right)}\right)=\mathrm{ReLU}\left(W^{\left(l+1\right)}a^{\left(l\right)}+b^{l+1}\right)$$

(2) ReLUx=x, if x>00, if x≤0

Moreover, the resulting feature matrix $a^{\left(l+1\right)}$ undergoes batch normalization and is combined with the sequence features to create a novel feature matrix that is inputted into a fully connected layer. This layer is followed by a Sigmoid activation function to generate the prediction outcomes. Thus, the process of predicting the probability of sample $S_{i}$ being an eRNA by utilizing the aforementioned operations can be expressed as follows:


(3) $$\mathrm{eRNAProb}\left(S_{i}\right)=\mathrm{Sigmoid}\left(\mathrm{Dense}\left(\left[BN\left(a^{\left(l+1\right)}\right),S_{\mathrm{feature}}\right]\right)\right)$$

### 2.4 Model training

To determine the hyperparameters in DeepITEH, such as epoch, batch size, and number of hidden cells, a 5-fold cross-validation and grid search strategy is used. Grid search can find global optimal hyperparameter values by traversing all possible combinations, which improves model stability and generalization. Combining it with cross-validation can further enhance search accuracy. The training set of each tissue is first divided into five subsets that are consistent in number and independent of each other. Then, for a given set of hyperparameters, one subset is selected as the validation set for evaluating the hyperparameters, and the remaining four subsets are used as the training data set for model training. The process is repeated five times, and the five validation results are averaged to evaluate the set of hyperparameters and ultimately obtain the best hyperparameter settings (Supplementary Tables S10 and S11).

Once the optimal hyperparameters of the model are obtained through the 5-fold cross-validation and grid search strategy, the final model is trained using the original training set data (i.e. training set + validation set) accordingly. The binary cross-entropy loss function is utilized as the objective function during the model training process, i.e. where $S_{i}$ denotes the ith sample, $y_{i}$ denotes its corresponding true label, and N denotes the total number of training samples. Specifically, the training set is divided into batches, and the binary cross-entropy loss function is used to calculate the loss values for each sample within a batch. The average loss value for all samples within a batch is then used to update the model parameters via the optimizer, and this process is repeated until the maximum number of iterations is achieved. The Adam (Kingma and Ba 2014) optimizer is used to optimize the training loss of the model. The Adam optimizer utilizes a combination of momentum and adaptive learning rate adjustment, leading to fast convergence and avoidance of overfitting. Additionally, Adam is robust to parameter initialization, resulting in an efficient and stable training process.


(4) $$\mathrm{Loss}=-\frac{1}{N}\sum_{i=1}^{N}y_{i}\log\;\mathrm{Prob}\left(S_{i}\right)+\left(1-y_{i}\right)\log\left(1-\mathrm{Prob}\left(S_{i}\right)\right)$$

### 2.5 Model evaluation

In this study, four standard evaluation metrics are utilized to evaluate the performance of DeepITEH. These evaluation metrics include accuracy (ACC), sensitivity (SN), specificity (SP), and Mathews correlation coefficient (MCC). The definitions of these evaluation metrics are as follows: where TP, TN, FP, and FN represent true positive, true negative, false positive, and false negative, respectively. Furthermore, receiver–operating characteristic (ROC) curves and the area under the curve (AUC) are included as performance indicators to better evaluate the overall performance of DeepITEH. Subsequently, we assess the performance of DeepITEH using independent datasets from each tissue, based on the specified performance metrics.


(5) $$\mathrm{SN}=\frac{\mathrm{TP}}{\mathrm{TP}+\mathrm{FN}}$$

(6) $$\mathrm{SP}=\frac{\mathrm{TN}}{\mathrm{TN}+\mathrm{FP}}$$

(7) $$\mathrm{ACC}=\frac{\mathrm{TP}+\mathrm{TN}}{\mathrm{TP}+\mathrm{TN}+\mathrm{FP}+\mathrm{FN}}$$

(8) $$\mathrm{MCC}=\frac{\mathrm{TP}\times \mathrm{TN}-\mathrm{FP}\times \mathrm{FN}}{\sqrt{\left(\mathrm{TP}+\mathrm{FP}\right)\left(\mathrm{FP}+\mathrm{FN}\right)\left(\mathrm{TN}+\mathrm{FP}\right)\left(\mathrm{TN}+\mathrm{FN}\right)}}$$

## 3 Results

### 3.1 Sequence features at the middle position of enhancer RNA are most obvious

**Figure 6:**
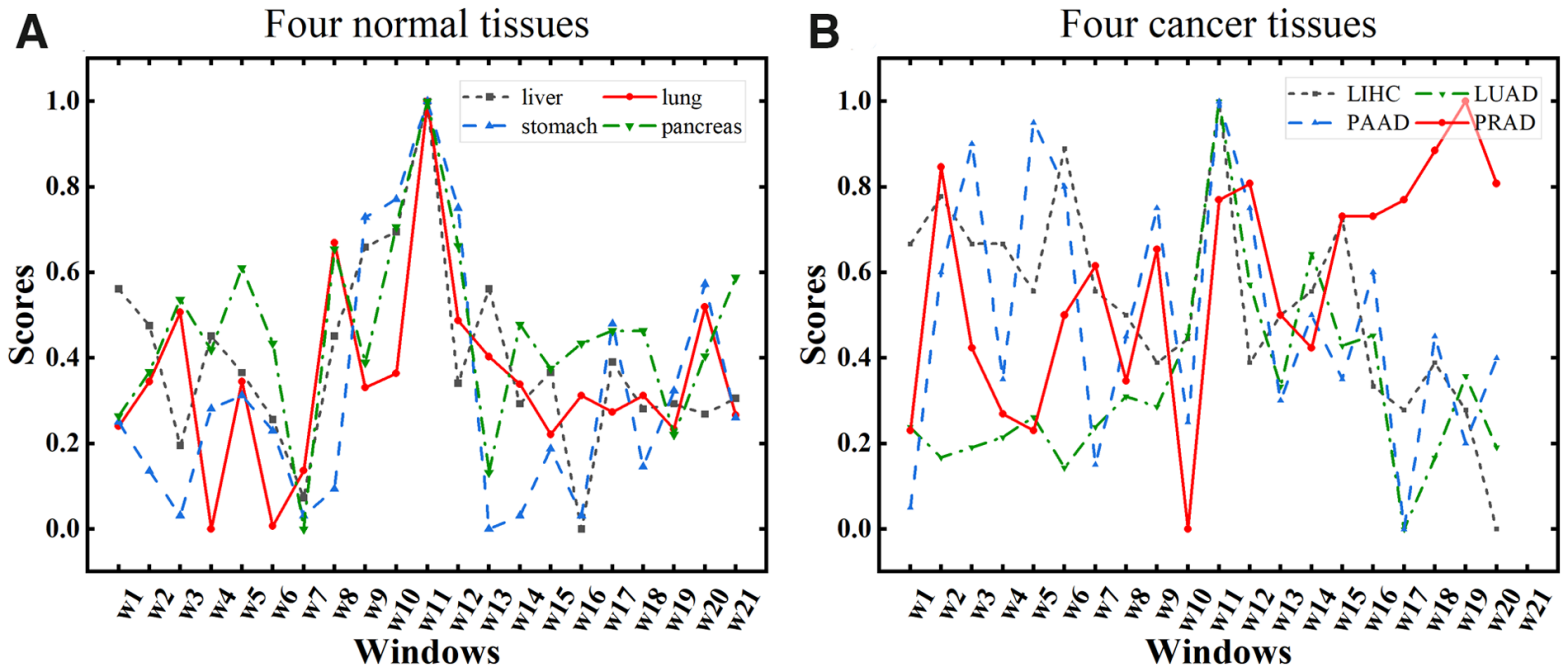
Comparison of sequence features of different windows of eRNA in the same tissue. (A) The scores of each window of eRNA in stomach, lung, liver and pancreas tissues. (B) The scores of each window of eRNA in liver-hepatocellular-carcinoma (LIHC), lung-adenocarcinoma (LUAD), Pancreatic-adenocarcinoma (PAAD), and prostate-adenocarcinoma (PRAD) tissues.

The window size used for dividing the eRNA is determined based on the average length of enhancers in the FANTOM database, which is 300 base pairs. The eRNA sequence is 6001 base pairs in length and is divided into 21 windows using this window size. The sequence features of each window are assigned scores using a scorer, and the window with the highest score is selected for extracting the sequence features of the eRNA. Figure 6A depicts the 21 window scores of eRNA in the four normal tissues, with the optimal windows being the middle window (w11). Figure 6B shows the 21 window scores of eRNA in the four cancer tissues, with the exception of PRAD tissue, where the optimal windows are the middle windows (w11). As a result, the middle position of the eRNA has the most prominent sequence features in most tissues. Moreover, extending 3000 bp upstream and downstream from the enhancer's middle base in FANTOM produces the eRNA. The middle region of eRNA is the original enhancer region, and the experimental results also confirm that the middle window of the eRNA has the highest sequence feature score in most tissues, validating the effectiveness and rationality of our approach to extract sequence features. In conclusion, sequence features are extracted from the middle window of eRNA in each tissue.

### 3.2 Histone modification features at the middle position of enhancer RNA are most obvious

Determining the location of histone modification features of eRNAs requires a comprehensive consideration of the definition of enhancers in three databases of eRNA sources. First, the average lengths of the peaks in each type of histone modification data for different samples in eight tissues are obtained as Supplementary Tables S12–S19. Then, the window size for extracting each type of histone modification feature in each tissue is determined according to the rules for dividing eRNA, as shown in Supplementary Tables S20–S27. The eRNAs in each tissue are then divided according to different window sizes, and the strength of the same histone modification features on their different windows is compared based on the method of selecting the optimal window, as shown in Supplementary Figs. S1–S8. By comparing each window, the optimal windows in the four normal tissues and the four cancer tissues are obtained as shown in Supplementary Table S28 and Table 1. From the table, 95% of the optimal windows in the four normal tissues are in and around the middle window (Mw and Aw), and only 5% of the optimal windows are far from the middle window (Fw); 90% of the optimal windows in the four cancer tissues are in and around the middle window, and 10% of the optimal windows are far from the middle window. Additionally, most of the optimal windows away from the middle window in the eight tissues are used to extract H3K36me3 features, indicating that H3K36me3 of eRNA is more complex. In conclusion, the optimal window for extracting each type of histone modification features in each tissue is the middle window or near the middle window, indicating that the histone modification features in the middle region of eRNA are the most obvious and overlap with the original enhancer region of eRNA, and justifying the rationality of our method for extracting histone modification features.

**Table 1.** Optimal windows for extraction of each type of histone modification features in four cancer tissues.

Tissues	LIHC	LUAD	PRAD	PAAD
H3K4me1	Mw	Mw	Mw	Mw
H3K4me3	Mw	Mw	Mw	Mw
H3K9me3	Mw	Aw	Mw	Aw
H3K27ac	Mw	Mw	Mw	Mw
H3K36me3	Aw	Aw	Fw	Fw

### 3.3 Different proportions of regularly expressed enhancer RNAs and accidentally expressed enhancer RNAs in different tissues

**Figure 7:**
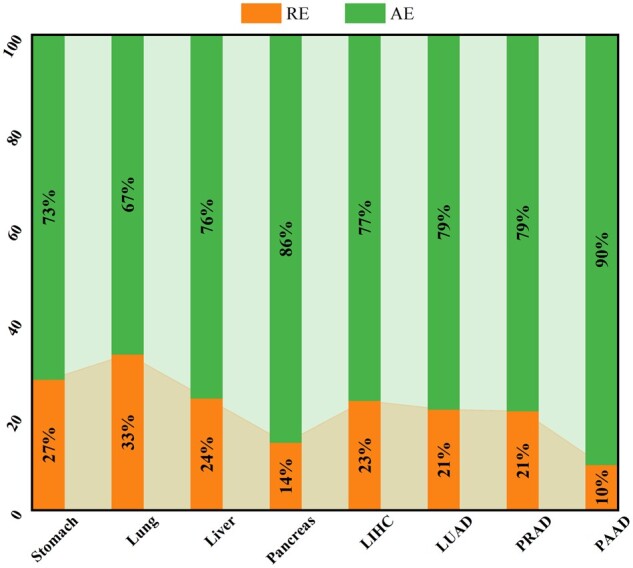
Proportions of RE and AE in different tissues.

The proportions of RE and AE are analyzed in four normal tissues and candidate REs and AEs in different samples in each tissue, as shown in Supplementary Tables S29–S32. The results indicate that the proportion of RE is relatively high in stomach, lung, and liver tissues, with values above 24%, while it is only 14% in pancreas tissue, as depicted in Fig. 7. Furthermore, we observed that DeepITEH is more effective in identifying eRNAs in independent test sets of stomach, lung, and liver tissues than in pancreas tissue, as demonstrated in Supplementary Fig. S9A. These findings suggest that the histone modifications of eRNAs in pancreas tissue are more complex, resulting in fewer REs. Additionally, in the four cancer tissues, the percentage of RE in LIHC, LUAD, and PRAD tissues are all above 21%, while the percentage of RE in PAAD tissue is only 10%, as presented in Supplementary Table S33 and Fig. 7. Similarly, DeepITEH performed better in identifying eRNAs in independent test sets of LIHC, LUAD, and PRAD tissues than in PAAD tissue, as shown in Supplementary Fig. S9B. Our results suggest that the histone modifications of eRNAs in different tissues vary, leading to differences in the proportions of RE. Specifically, for pancreatic tissues (normal and cancer), the histone modifications of eRNAs are complex, resulting in relatively few REs.

### 3.4 Compare the accuracy of enhancer RNA identification based on different samples in the same tissue

**Figure 8:**
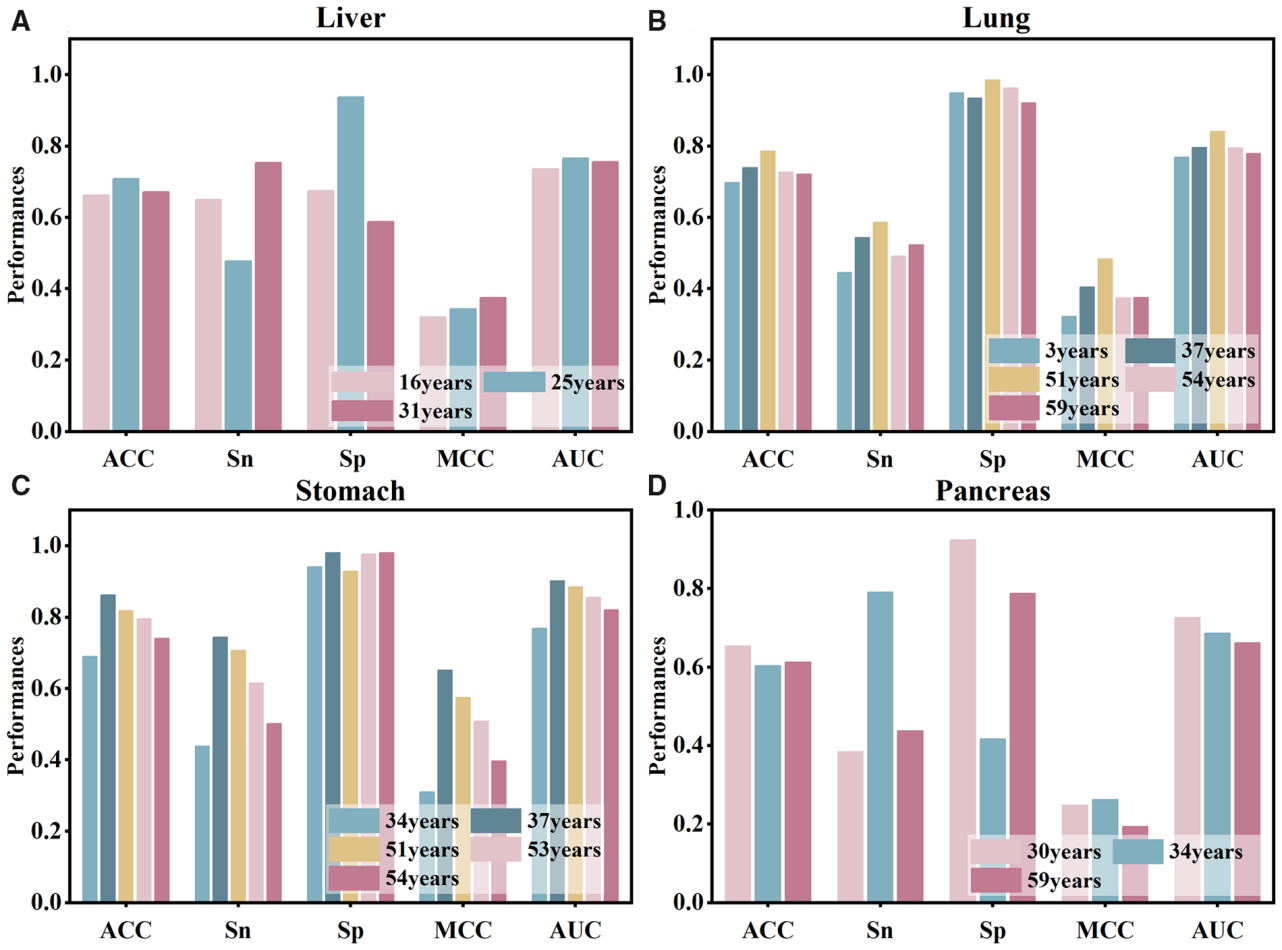
Comparison of the accuracy of different samples in the same tissue for identifying eRNA. (A) Comparison of different samples in the liver tissue on five metrics. (B) Comparison of different samples in the lung tissue on five metrics. (C) Comparison of different samples in the stomach tissue on five metrics. (D) Comparison of different samples in the pancreas tissue on five metrics.

In this study, we utilize histone modification data from different samples of the same tissue, ensuring that at least one sample of each tissue has such data. For tissues with multiple samples, we construct models to recognize eRNA based on each sample separately. We assess the performance of the models using five metrics to evaluate the accuracy of different samples in identifying eRNA, and then select the sample used in the optimal model. Figure 8A compares the accuracy of different samples in liver tissue to identify eRNA, where sample 25 years old demonstrates higher scores in three indicators (ACC, Sp, and AUC) than other samples, and is hence selected to construct the prediction model of eRNA in liver tissue. Figure 8B compares the accuracy of different samples in lung tissue for identifying eRNA, where sample 51 years old outperforms all other samples in all five indexes and is therefore selected to construct a prediction model for eRNA in lung tissue. Figure 8C displays the accuracy comparison of different samples in stomach tissue for identifying eRNA, wherein sample 37 years old outperforms other samples in all five performance indexes, and hence is chosen to construct the prediction model of eRNA in stomach tissue. Additionally, Fig. 8D compares the accuracy of eRNA identification in different samples in pancreas tissue, where sample 30 years old exhibits higher scores in three indexes (ACC, Sp, and AUC) than other samples, and is thus selected to construct the prediction model of eRNA in pancreas tissue. Lastly, since LIHC, LUAD, PRAD, and PAAD tissues each have only one sample, we construct the prediction models for eRNA in each tissue based on their respective samples.

### 3.5 Contribution of postfusion features compared with using only sequence features to predict enhancer RNA

**Figure 9:**
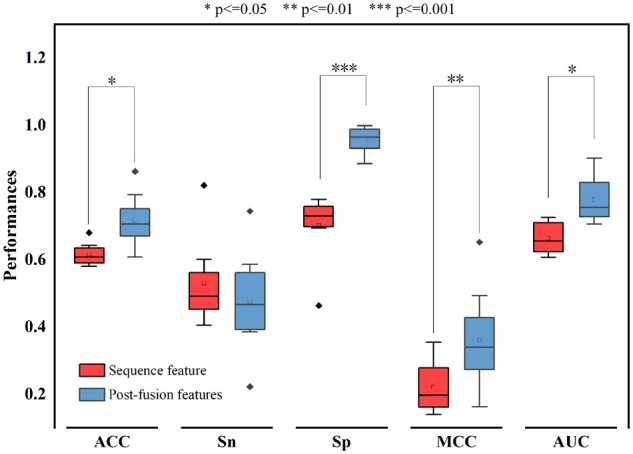
Contribution of postfusion features compared with using only sequence features to predict eRNA.

In this section, the aim is to evaluate the impact of postfusion features and showcase the enhancement of DeepITEH in accurately identifying tissue-specific eRNAs using DNA sequence data alone. To achieve this, a comparison between postfusion features and sequence-only features is conducted on an independent test set of eight different tissues using five metrics. Results indicate that the postfusion features outperform sequence-only features on all four metrics, including ACC, Sp, MCC, and AUC, as illustrated in Fig. 9. Furthermore, a double overall *t*-test with *P*-value ≤0.05 is used to demonstrate the significant difference between the means of the two samples. The differences are significant in all four indicators, ACC, Sp, MCC, and AUC, as shown in Fig. 9. Overall, the postfusion features provide more predictive information than using sequence features alone, substantially improving the accuracy of predicting tissue-specific eRNA.

### 3.6 DeepITEH compared with state-of-the-art prediction methods

**Figure 10:**
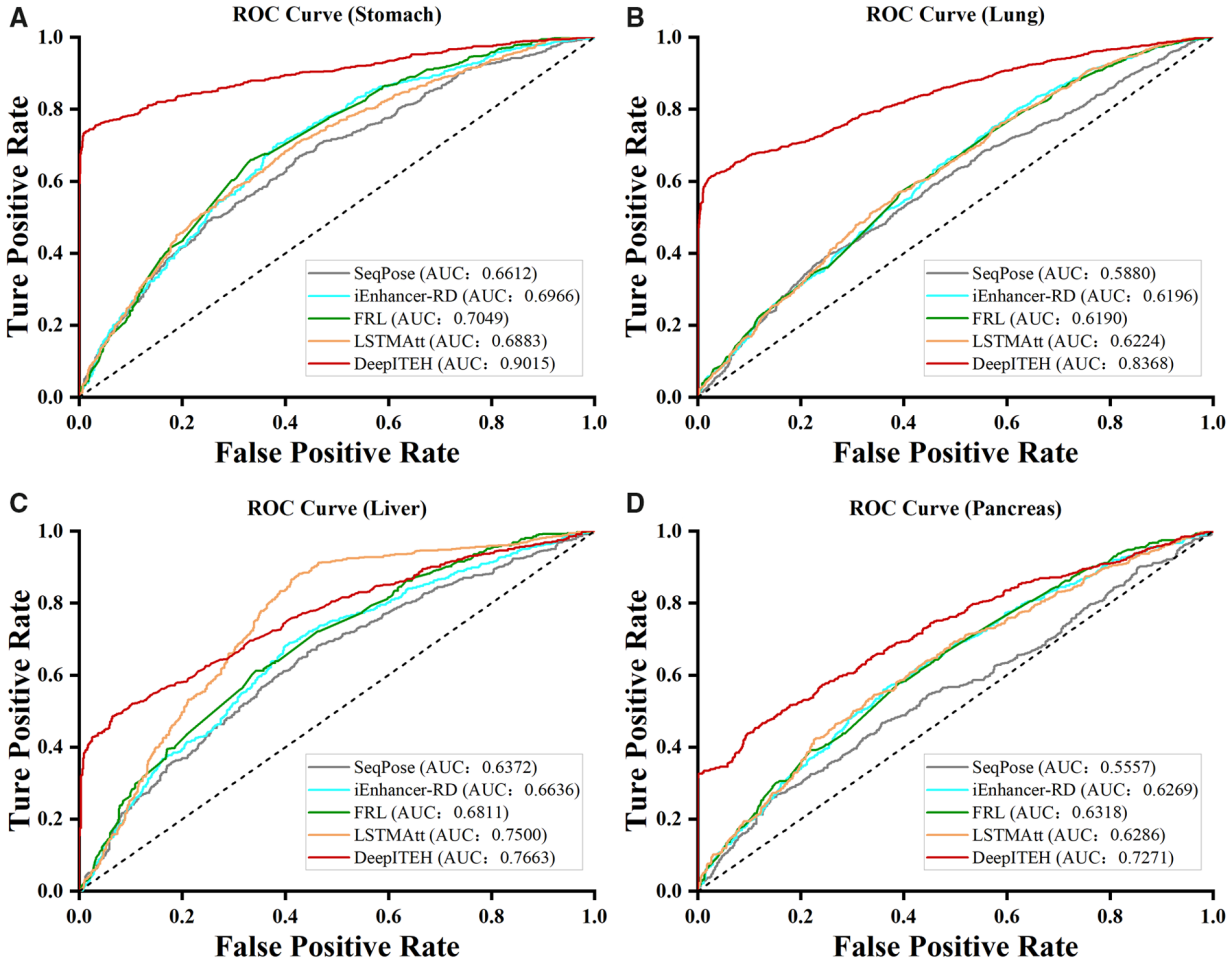
Receiver operating characteristic (ROC) curves and area under the curve (AUC) for DeepITEH and state-of-the-art prediction methods in four normal tissues, namely stomach, lung, liver, and pancreas, are shown in Figures A, B, C, and D, respectively.

In order to demonstrate the accuracy of eRNA identification in normal tissues using DeepITEH, we conducted a comparative analysis with four prediction methods that rely solely on sequence features, namely, the SeqPose, iEnhancer-RD, LSTMAtt, and FRL. This analysis was performed using five evaluation metrics on independent datasets of four normal tissues. Our results indicated that the ACC metrics of DeepITEH were consistently superior to those of the other methods across different independent test sets, as presented in Table 2. Notably, DeepITEH exhibited superior performance across all five evaluation metrics on the independent test set of gastric tissue, as demonstrated in Supplementary Table S34. Additionally, DeepITEH outperformed the other methods across four evaluation metrics on the independent test sets of lung, liver, and pancreas tissues, as shown in Supplementary Tables S35–S37. The ROC curves for the four normal tissues were plotted and displayed in Fig. 10. These results indicate that DeepITEH performs well on all four independent test sets of normal tissues, effectively improving the accuracy of identifying tissue-specific eRNAs. On the other hand, the other four prediction methods generally exhibited low performance in different tissues, suggesting that they overlooked the tissue specificity of eRNAs, leading to poor accuracy in identifying eRNAs in specific tissues.

**Table 2.** Comparison of models on independent test sets of four normal tissues.

Method	Stomach	Lung	Liver	Pancreas
	ACC (%)	ACC (%)	ACC (%)	ACC (%)
SeqPose	61.09	56.37	58.75	55.12
iEnhancer-RD	63.88	57.18	60.80	58.53
FRL	66.22	58.18	62.78	58.98
LSTMAtt	63.94	58.80	65.85	59.42
DeepITEH	**86.25**	**78.59**	**70.74**	**65.43**

Boldface values indicate the highest ACC value in each column.

**Figure 11:**
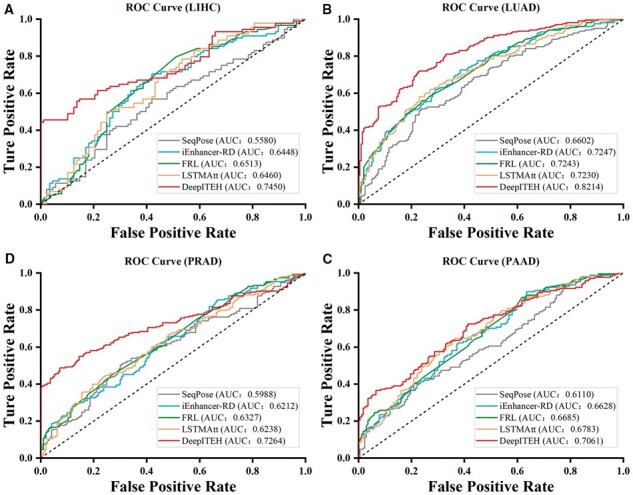
Receiver operating characteristic (ROC) curves and area under the curve (AUC) for DeepITEH and state-of-the-art prediction methods in four cancer tissues, namely liver-hepatocellular-carcinoma(LIHC), lung-adenocarcinoma(LUAD), prostate-adenocarcinoma(PRAD) and Pancreatic-adenocarcinoma(PAAD), are shown in Figures A, B, C, and D, respectively.

To verify the effectiveness of DeepITEH in identifying eRNAs in cancer tissues, we conducted a comparative analysis between DeepITEH and the four aforementioned methods, using independent datasets of four cancer tissues. Our results revealed that, with the exception of PAAD, DeepITEH consistently demonstrated higher ACC scores than the other methods across the independent test sets of the remaining three cancer tissues, as presented in Table 3. Specifically, DeepITEH exhibited superior performance across four evaluation metrics in the independent test sets of LIHC, LUAD, and PRAD tissues, as illustrated in Supplementary Tables S38–S40. On the independent test set of PAAD tissue, DeepITEH outperforms the other methods in two evaluation metrics, as shown in Supplementary Table S41. Although the model's ACC for predicting eRNAs in PAAD tissue is low due to the more complex histone modifications in that tissue, the performance of DeepITEH is still better than other prediction methods based on the evaluation index AUC. The ROC curves on the independent test sets of the four cancer tissues are shown in Fig. 11. Our results indicate that DeepITEH performs well in all four cancer tissues, demonstrating high robustness in LIHC, LUAD, and PRAD tissues. By effectively improving the accuracy of identifying tissue-specific eRNAs, DeepITEH outperforms the other four methods, which generally have low performance, suggesting that these methods similarly overlook the tissue specificity of eRNAs in cancer tissues, resulting in poor accuracy in identifying eRNAs in specific cancer tissues.

**Table 3.** Comparison of models on independent test sets of four cancer tissues.

Method	LIHC	LUAD	PRAD	PAAD
	ACC (%)	ACC (%)	ACC (%)	ACC (%)
SeqPose	55.11	59.00	58.81	57.19
iEnhancer-RD	61.36	65.25	55.44	59.88
FRL	61.93	65.75	57.51	60.18
LSTMAtt	58.52	66.50	58.55	**63.47**
DeepITEH	**70.45**	**71.00**	**68.65**	60.78

Boldface values indicate the highest ACC value in each column.

## 4 Discussion

Our study introduces a novel deep learning framework, called DeepITEH, for accurate prediction of eRNAs. DeepITEH combines RNA-seq data with histone modification data from various samples of the same tissue within a deep learning framework. Extensive experiments conducted in different tissues demonstrate that DeepITEH is highly effective in improving the accuracy of eRNA prediction for specific tissues. Furthermore, our proposed method outperforms the current state-of-the-art methods, namely, SeqPose, iEnhancer-RD, LSTMAtt, and FRL, on independent test sets of both normal and cancer tissues in terms of ROAUC. Based on our findings, we conclude that DeepITEH is a precise, stable, and advanced prediction method capable of accurately identifying potential eRNAs from the human genome.

Further exploration of DeepITEH may lead to promising improvements in enhancer identification. First, integrating additional data sources such as ChIP-seq data for transcription factors, ChIP-seq data for the transcriptional coactivator p300, and chromatin accessibility data may enhance the accuracy of enhancer identification, as evidenced by some previous studies. Second, DeepITEH could be extended to predict enhancers that are not associated with transcriptional activity. Third, recent research has shed light on enhancer action sites, and the eRNA feature extraction method used in this study could be applied to promoters to facilitate the construction of associations between enhancers and promoters.

## Supplementary Material

btad375_Supplementary_Data

## Data Availability

The source code and dataset of DeepITEH have been uploaded to https://github.com/lyli1013/DeepITEH.
