# Supplementary material for: DeepITEH: a deep learning framework for identifying tissue-specific eRNAs from the human genome
Source: Bioinformatics. 2023 Jun 9;39(6):btad375. doi: 10.1093/bioinformatics/btad375 (PMC10281860; doi:10.1093/bioinformatics/btad375)
Supplement: btad375_Supplementary_Data [file btad375_supplementary_data.zip › Supplementary file.docx]

**Table S1.** Sample size of the datasets for different tissue types

| **Tissue** | **No. of eRNA** | **No. of NE** | **Training** | **Testing** |
| --- | --- | --- | --- | --- |
| Stomach | 3943 | 3943 | 6308 | 1578 |
| Lung | 6481 | 6481 | 1,0370 | 2592 |
| Liver | 3171 | 3171 | 5074 | 1268 |
| Pancreas | 3368 | 3368 | 5388 | 1348 |
| LIHC | 442 | 442 | 708 | 176 |
| LUAD | 1178 | 1178 | 1956 | 400 |
| PRAD | 964 | 964 | 1542 | 386 |
| PAAD | 835 | 835 | 1336 | 334 |

**Table S2.** Histone modification data of different samples of stomach tissues.

| Stomach | H3K4me1 | H3K4me3 | H3K9me3 | H3K27ac | H3K36me3 |
| --- | --- | --- | --- | --- | --- |
| 34years | [ENCSR257BCD](https://www.encodeproject.org/experiments/ENCSR257BCD/) | [ENCSR129NCV](https://www.encodeproject.org/experiments/ENCSR129NCV/) | [ENCSR639RKZ](https://www.encodeproject.org/experiments/ENCSR639RKZ/) | [ENCSR001SHB](https://www.encodeproject.org/experiments/ENCSR001SHB/) | [ENCSR552MZH](https://www.encodeproject.org/experiments/ENCSR552MZH/) |
| 37years | [ENCSR493MQY](https://www.encodeproject.org/experiments/ENCSR493MQY/) | [ENCSR843UEZ](https://www.encodeproject.org/experiments/ENCSR843UEZ/) | [ENCSR503XFW](https://www.encodeproject.org/experiments/ENCSR503XFW/) | [ENCSR944KAZ](https://www.encodeproject.org/experiments/ENCSR944KAZ/) | [ENCSR552ICY](https://www.encodeproject.org/experiments/ENCSR552ICY/) |
| 51years | [ENCSR009RJD](https://www.encodeproject.org/experiments/ENCSR009RJD/) | [ENCSR492BHN](https://www.encodeproject.org/experiments/ENCSR492BHN/) | [ENCSR787TQW](https://www.encodeproject.org/experiments/ENCSR787TQW/) | [ENCSR751BHO](https://www.encodeproject.org/experiments/ENCSR751BHO/) | [ENCSR819DOR](https://www.encodeproject.org/experiments/ENCSR819DOR/) |
| 53years | [ENCSR903QBX](https://www.encodeproject.org/experiments/ENCSR903QBX/) | [ENCSR489ZLL](https://www.encodeproject.org/experiments/ENCSR489ZLL/) | [ENCSR546HZF](https://www.encodeproject.org/experiments/ENCSR546HZF/) | [ENCSR133NBJ](https://www.encodeproject.org/experiments/ENCSR133NBJ/) | [ENCSR166CNR](https://www.encodeproject.org/experiments/ENCSR166CNR/) |
| 54years | [ENCSR158WBG](https://www.encodeproject.org/experiments/ENCSR158WBG/) | [ENCSR063HOI](https://www.encodeproject.org/experiments/ENCSR063HOI/) | [ENCSR475TRZ](https://www.encodeproject.org/experiments/ENCSR475TRZ/) | [ENCSR204OJS](https://www.encodeproject.org/experiments/ENCSR204OJS/) | [ENCSR007GED](https://www.encodeproject.org/experiments/ENCSR007GED/) |

**Table S3.** Histone modification data of different samples of lung tissues.

| Lung | H3K4me1 | H3K4me3 | H3K9me3 | H3K27ac | H3K36me3 |
| --- | --- | --- | --- | --- | --- |
| 3years | [ENCSR575SWA](https://www.encodeproject.org/experiments/ENCSR575SWA/) | [ENCSR500GXT](https://www.encodeproject.org/experiments/ENCSR500GXT/) | [ENCSR456TZO](https://www.encodeproject.org/experiments/ENCSR456TZO/) | [ENCSR550WUX](https://www.encodeproject.org/experiments/ENCSR550WUX/) | [ENCSR429MNF](https://www.encodeproject.org/experiments/ENCSR429MNF/) |
| 37years | [ENCSR595MTV](https://www.encodeproject.org/experiments/ENCSR595MTV/) | [ENCSR074WIB](https://www.encodeproject.org/experiments/ENCSR074WIB/) | [ENCSR714FDJ](https://www.encodeproject.org/experiments/ENCSR714FDJ/) | [ENCSR505YFA](https://www.encodeproject.org/experiments/ENCSR505YFA/) | [ENCSR276OPI](https://www.encodeproject.org/experiments/ENCSR276OPI/) |
| 51years | [ENCSR238WIK](https://www.encodeproject.org/experiments/ENCSR238WIK/) | [ENCSR429VWL](https://www.encodeproject.org/experiments/ENCSR429VWL/) | [ENCSR623YMO](https://www.encodeproject.org/experiments/ENCSR623YMO/) | [ENCSR453MUW](https://www.encodeproject.org/experiments/ENCSR453MUW/) | [ENCSR642DSR](https://www.encodeproject.org/experiments/ENCSR642DSR/) |
| 54years | [ENCSR348FGT](https://www.encodeproject.org/experiments/ENCSR348FGT/) | [ENCSR701FGA](https://www.encodeproject.org/experiments/ENCSR701FGA/) | [ENCSR038ODI](https://www.encodeproject.org/experiments/ENCSR038ODI/) | [ENCSR948TOS](https://www.encodeproject.org/experiments/ENCSR948TOS/) | [ENCSR229GEL](https://www.encodeproject.org/experiments/ENCSR229GEL/) |
| 59years | [ENCSR155EOT](https://www.encodeproject.org/experiments/ENCSR155EOT/) | [ENCSR791BWS](https://www.encodeproject.org/experiments/ENCSR791BWS/) | [ENCSR368APT](https://www.encodeproject.org/experiments/ENCSR368APT/) | [ENCSR313CEH](https://www.encodeproject.org/experiments/ENCSR313CEH/) | [ENCSR152VOJ](https://www.encodeproject.org/experiments/ENCSR152VOJ/) |

**Table S4.** Histone modification data of different samples of liver tissues.

| Liver | H3K4me1 | H3K4me3 | H3K9me3 | H3K27ac | H3K36me3 |
| --- | --- | --- | --- | --- | --- |
| 16years | [ENCSR554XXQ](https://www.encodeproject.org/experiments/ENCSR554XXQ/) | [ENCSR786ALM](https://www.encodeproject.org/experiments/ENCSR786ALM/) | [ENCSR590AJV](https://www.encodeproject.org/experiments/ENCSR590AJV/) | [ENCSR119XNK](https://www.encodeproject.org/experiments/ENCSR119XNK/) | [ENCSR423SEX](https://www.encodeproject.org/experiments/ENCSR423SEX/) |
| 25years | [ENCSR642HII](https://www.encodeproject.org/experiments/ENCSR642HII/) | [ENCSR803JYI](https://www.encodeproject.org/experiments/ENCSR803JYI/) | [ENCSR853JYB](https://www.encodeproject.org/experiments/ENCSR853JYB/) | [ENCSR678LND](https://www.encodeproject.org/experiments/ENCSR678LND/) | [ENCSR213DKS](https://www.encodeproject.org/experiments/ENCSR213DKS/) |
| 31years | [ENCSR111OHT](https://www.encodeproject.org/experiments/ENCSR111OHT/) | [ENCSR458WIH](https://www.encodeproject.org/experiments/ENCSR458WIH/) | [ENCSR351DLF](https://www.encodeproject.org/experiments/ENCSR351DLF/) | [ENCSR230IMS](https://www.encodeproject.org/experiments/ENCSR230IMS/) | [ENCSR460RHN](https://www.encodeproject.org/experiments/ENCSR460RHN/) |

**Table S5.** Histone modification data of different samples of pancreas tissues.

| Pancreas | H3K4me1 | H3K4me3 | H3K9me3 | H3K27ac | H3K36me3 |
| --- | --- | --- | --- | --- | --- |
| 30years | [ENCSR984UHU](https://www.encodeproject.org/experiments/ENCSR984UHU/) | [ENCSR315LPR](https://www.encodeproject.org/experiments/ENCSR315LPR/) | [ENCSR533HDU](https://www.encodeproject.org/experiments/ENCSR533HDU/) | [ENCSR402HFW](https://www.encodeproject.org/experiments/ENCSR402HFW/) | [ENCSR393HBQ](https://www.encodeproject.org/experiments/ENCSR393HBQ/) |
| 34years | [ENCSR449PYI](https://www.encodeproject.org/experiments/ENCSR449PYI/) | [ENCSR747VED](https://www.encodeproject.org/experiments/ENCSR747VED/) | [ENCSR035QNZ](https://www.encodeproject.org/experiments/ENCSR035QNZ/) | [ENCSR612BWE](https://www.encodeproject.org/experiments/ENCSR612BWE/) | [ENCSR943JOF](https://www.encodeproject.org/experiments/ENCSR943JOF/) |
| 59years | [ENCSR233EDV](https://www.encodeproject.org/experiments/ENCSR233EDV/) | [ENCSR713YDD](https://www.encodeproject.org/experiments/ENCSR713YDD/) | [ENCSR368EPJ](https://www.encodeproject.org/experiments/ENCSR368EPJ/) | [ENCSR868ZOR](https://www.encodeproject.org/experiments/ENCSR868ZOR/) | [ENCSR637NGB](https://www.encodeproject.org/experiments/ENCSR637NGB/) |

**Table S6.** Histone modification data of LIHC tissue.

| LIHC | H3K4me1 | H3K4me3 | H3K9me3 | H3K27ac | H3K36me3 |
| --- | --- | --- | --- | --- | --- |
| HeG2 | [ENCSR000APV](https://www.encodeproject.org/experiments/ENCSR000APV/) | [ENCSR000AMP](https://www.encodeproject.org/experiments/ENCSR000AMP/) | [ENCSR000ATD](https://www.encodeproject.org/experiments/ENCSR000ATD/) | [ENCSR000AMO](https://www.encodeproject.org/experiments/ENCSR000AMO/) | [ENCSR000AMB](https://www.encodeproject.org/experiments/ENCSR000AMB/) |

**Table S7.** Histone modification data of LUAD tissue.

| LUAD | H3K4me1 | H3K4me3 | H3K9me3 | H3K27ac | H3K36me3 |
| --- | --- | --- | --- | --- | --- |
| PC-9 | [ENCSR913MGR](https://www.encodeproject.org/experiments/ENCSR913MGR/) | [ENCSR441JWF](https://www.encodeproject.org/experiments/ENCSR441JWF/) | [ENCSR555TAX](https://www.encodeproject.org/experiments/ENCSR555TAX/) | [ENCSR769FOC](https://www.encodeproject.org/experiments/ENCSR769FOC/) | [ENCSR402SJV](https://www.encodeproject.org/experiments/ENCSR402SJV/) |

**Table S8.** Histone modification data of PRAD tissue.

| PRAD | H3K4me1 | H3K4me3 | H3K9me3 | H3K27ac | H3K36me3 |
| --- | --- | --- | --- | --- | --- |
| PC-3 | [ENCSR566UMF](https://www.encodeproject.org/experiments/ENCSR566UMF/) | [ENCSR275NCH](https://www.encodeproject.org/experiments/ENCSR275NCH/) | [ENCSR339ZMJ](https://www.encodeproject.org/experiments/ENCSR339ZMJ/) | [ENCSR826UTD](https://www.encodeproject.org/experiments/ENCSR826UTD/) | [ENCSR849APH](https://www.encodeproject.org/experiments/ENCSR849APH/) |

**Table S9.** Histone modification data of PAAD tissue.

| PAAD | H3K4me1 | H3K4me3 | H3K9me3 | H3K27ac | H3K36me3 |
| --- | --- | --- | --- | --- | --- |
| Panc1 | [ENCSR000EXJ](https://www.encodeproject.org/experiments/ENCSR000EXJ/) | [ENCSR000EXI](https://www.encodeproject.org/experiments/ENCSR000EXI/) | [ENCSR000FCN](https://www.encodeproject.org/experiments/ENCSR000FCN/) | [ENCSR000EXK](https://www.encodeproject.org/experiments/ENCSR000EXK/) | [ENCSR000FCM](https://www.encodeproject.org/experiments/ENCSR000FCM/) |

**Table S10.** Determination of optimal hyperparameters for eRNA identification models in specific normal tissues using a grid search strategy.

| Hyperparamter | Stomach | Lung | Liver | Pancreas |
| --- | --- | --- | --- | --- |
| epoch | 10 | 8 | 10 | 8 |
| batch size | 16 | 64 | 64 | 16 |
| dropout (LSTM) | 0.5 | 0.5 | 0.5 | 0.5 |
| dropout (DNN) | 0.1 | 0.1 | 0.1 | 0.1 |
| dense layers number | 2 | 2 | 2 | 2 |

**Table S11.** Determination of optimal hyperparameters for eRNA identification models in specific cancer tissues using a grid search strategy.

| Hyperparamter | LIHC | LUAD | PRAD | PAAD |
| --- | --- | --- | --- | --- |
| epoch | 9 | 4 | 4 | 7 |
| batch size | 4 | 64 | 64 | 32 |
| dropout (LSTM) | 0.5 | 0.5 | 0.5 | 0.5 |
| dropout (DNN) | 0.1 | 0.1 | 0.1 | 0.1 |
| dense layers number | 2 | 2 | 2 | 2 |

**Table S12.** Mean length of Peak in histone modification data of different samples of stomach tissue.

| Stomach | H3K4me1 | H3K4me3 | H3K9me3 | H3K27ac | H3K36me3 |
| --- | --- | --- | --- | --- | --- |
| 34years | 264 | 369 | 194 | 494 | 336 |
| 37years | 1114 | 1535 | 303 | 1216 | 1712 |
| 51years | 946 | 1340 | 262 | 1197 | 1563 |
| 53years | 676 | 1362 | 679 | 1270 | 2018 |
| 54years | 658 | 1318 | 382 | 1031 | 332 |

**Table S13.** Mean length of Peak in histone modification data of different samples of lung tissue.

| Lung | H3K4me1 | H3K4me3 | H3K9me3 | H3K27ac | H3K36me3 |
| --- | --- | --- | --- | --- | --- |
| 3years | 293 | 1006 | 220 | 452 | 293 |
| 37years | 567 | 1511 | 215 | 872 | 1698 |
| 51years | 843 | 1460 | 585 | 1085 | 373 |
| 54years | 731 | 1337 | 949 | 862 | 567 |
| 59years | 1176 | 1760 | 1031 | 1468 | 1502 |

**Table S14.** Mean length of Peak in histone modification data of different samples of liver tissue.

| Liver | H3K4me1 | H3K4me3 | H3K9me3 | H3K27ac | H3K36me3 |
| --- | --- | --- | --- | --- | --- |
| 16years | 1065 | 1505 | 717 | 1569 | 1283 |
| 25years | 519 | 331 | 241 | 682 | 376 |
| 31years | 449 | 76 | 279 | 879 | 526 |

**Table S15.** Mean length of Peak in histone modification data of different samples of pancreas tissue.

| Pancreas | H3K4me1 | H3K4me3 | H3K9me3 | H3K27ac | H3K36me3 |
| --- | --- | --- | --- | --- | --- |
| 30years | 222 | 1221 | 176 | 201 | 207 |
| 34years | 308 | 609 | 228 | 363 | 239 |
| 59years | 784 | 1797 | 559 | 1227 | 1219 |

**Table S16.** Mean length of Peak in histone modification data of LIHC tissue.

| LIHC | H3K4me1 | H3K4me3 | H3K9me3 | H3K27ac | H3K36me3 |
| --- | --- | --- | --- | --- | --- |
| HeG2 | 957 | 889 | 752 | 710 | 442 |

**Table S17.** Mean length of Peak in histone modification data of LUAD tissue.

| LUAD | H3K4me1 | H3K4me3 | H3K9me3 | H3K27ac | H3K36me3 |
| --- | --- | --- | --- | --- | --- |
| PC-9 | 1073 | 994 | 348 | 1024 | 254 |

**Table S18.** Mean length of Peak in histone modification data of PRAD tissue.

| PRAD | H3K4me1 | H3K4me3 | H3K9me3 | H3K27ac | H3K36me3 |
| --- | --- | --- | --- | --- | --- |
| PC-3 | 712 | 935 | 380 | 972 | 509 |

**Table S19.** Mean length of Peak in histone modification data of PAAD tissue.

| PAAD | H3K4me1 | H3K4me3 | H3K9me3 | H3K27ac | H3K36me3 |
| --- | --- | --- | --- | --- | --- |
| Panc1 | 590 | 1568 | 403 | 918 | 259 |

**Table S20.** For eRNA in stomach tissue, the window size for extracting its histone modification features of each type is as follows.

| Stomach | H3K4me1 | H3K4me3 | H3K9me3 | H3K27ac | H3K36me3 |
| --- | --- | --- | --- | --- | --- |
| 34years | 300 | 400 | 300 | 500 | 400 |
| 37years | 1200 | 1600 | 400 | 1300 | 1800 |
| 51years | 1000 | 1400 | 300 | 1200 | 1600 |
| 53years | 700 | 1400 | 700 | 1300 | 2100 |
| 54years | 700 | 1400 | 400 | 1100 | 400 |

**Table S21.** For eRNA in lung tissue, the window size for extracting its histone modification features of each type is as follows.

| Lung | H3K4me1 | H3K4me3 | H3K9me3 | H3K27ac | H3K36me3 |
| --- | --- | --- | --- | --- | --- |
| 3years | 300 | 1100 | 300 | 500 | 300 |
| 37years | 600 | 1600 | 300 | 900 | 1700 |
| 51years | 900 | 1500 | 600 | 1100 | 400 |
| 54years | 800 | 1400 | 1000 | 900 | 600 |
| 59years | 1200 | 1800 | 1100 | 1500 | 1600 |

**Table S22.** For eRNA in liver tissue, the window size for extracting its histone modification features of each type is as follows.

| Liver | H3K4me1 | H3K4me3 | H3K9me3 | H3K27ac | H3K36me3 |
| --- | --- | --- | --- | --- | --- |
| 16years | 1100 | 1600 | 800 | 1600 | 1300 |
| 25years | 600 | 400 | 300 | 700 | 400 |
| 31years | 500 | 800 | 300 | 900 | 600 |

**Table S23.** For eRNA in pancreas tissue, the window size for extracting its histone modification features of each type is as follows.

| Pancreas | H3K4me1 | H3K4me3 | H3K9me3 | H3K27ac | H3K36me3 |
| --- | --- | --- | --- | --- | --- |
| 30years | 300 | 1300 | 300 | 300 | 300 |
| 34years | 400 | 700 | 300 | 400 | 300 |
| 59years | 800 | 1800 | 600 | 1300 | 1300 |

**Table S24.** For eRNA in LIHC tissue, the window size for extracting its histone modification features of each type is as follows.

| LIHC | H3K4me1 | H3K4me3 | H3K9me3 | H3K27ac | H3K36me3 |
| --- | --- | --- | --- | --- | --- |
| HeG2 | 1000 | 900 | 800 | 800 | 500 |

**Table S25.** For eRNA in LUAD tissue, the window size for extracting its histone modification features of each type is as follows.

| LUAD | H3K4me1 | H3K4me3 | H3K9me3 | H3K27ac | H3K36me3 |
| --- | --- | --- | --- | --- | --- |
| PC-9 | 1100 | 1000 | 400 | 1100 | 300 |

**Table S26.** For eRNA in PRAD tissue, the window size for extracting its histone modification features of each type is as follows.

| PRAD | H3K4me1 | H3K4me3 | H3K9me3 | H3K27ac | H3K36me3 |
| --- | --- | --- | --- | --- | --- |
| PC-3 | 800 | 1000 | 400 | 1000 | 600 |

**Table S27.** For eRNA in PAAD tissue, the window size for extracting its histone modification features of each type is as follows.

| PAAD | H3K4me1 | H3K4me3 | H3K9me3 | H3K27ac | H3K36me3 |
| --- | --- | --- | --- | --- | --- |
| Panc1 | 600 | 1600 | 500 | 1000 | 300 |

**Figure S1.** For eRNA in stomach tissue, the comparison of the strength of the same type of histone modification features on its different windows. i, ii, iii, iv, and v denote stomach tissue samples from humans aged 34, 37, 51, 53, and 54 years, respectively. (A), (B), (C), (D) and (E) show the H3K4me1, H3K4me3, H3K9me3, H3K27ac and H3K36me3 features of eRNAs, respectively, where the horizontal coordinates denote each window of eRNAs and the vertical coordinates denote the number of eRNAs with the same histone modification feature on different windows.

**Figure S2.** For eRNA in lung tissue, the comparison of the strength of the same type of histone modification features on its different windows. i, ii, iii, iv, and v denote lung tissue samples from humans aged 3, 37, 51, 54, and 59 years, respectively. (A), (B), (C), (D) and (E) show the H3K4me1, H3K4me3, H3K9me3, H3K27ac and H3K36me3 features of eRNAs, respectively, where the horizontal coordinates denote each window of eRNAs and the vertical coordinates denote the number of eRNAs with the same histone modification feature on different windows.

**Figure S3.** For eRNA in liver tissue, the comparison of the strength of the same type of histone modification features on its different windows. i, ii and iii denote liver tissue samples from humans aged 16, 25 and 31 years, respectively. (A), (B), (C), (D) and (E) show the H3K4me1, H3K4me3, H3K9me3, H3K27ac and H3K36me3 features of eRNAs, respectively, where the horizontal coordinates denote each window of eRNAs and the vertical coordinates denote the number of eRNAs with the same histone modification feature on different windows.

**Figure S4.** For eRNA in pancreas tissue, the comparison of the strength of the same type of histone modification features on its different windows. i, ii and iii denote pancreas tissue samples from humans aged 30, 34 and 59 years, respectively. (A), (B), (C), (D) and (E) show the H3K4me1, H3K4me3, H3K9me3, H3K27ac and H3K36me3 features of eRNAs, respectively, where the horizontal coordinates denote each window of eRNAs and the vertical coordinates denote the number of eRNAs with the same histone modification feature on different windows.

**Figure S5.** For eRNA in LIHC tissue, the comparison of the strength of the same type of histone modification features on its different windows. (A), (B), (C), (D) and (E) show the H3K4me1, H3K4me3, H3K9me3, H3K27ac and H3K36me3 features of eRNAs, respectively, where the horizontal coordinates denote each window of eRNAs and the vertical coordinates denote the number of eRNAs with the same histone modification feature on different windows.

**Figure S6.** For eRNA in LUAD tissue, the comparison of the strength of the same type of histone modification features on its different windows. (A), (B), (C), (D) and (E) show the H3K4me1, H3K4me3, H3K9me3, H3K27ac and H3K36me3 features of eRNAs, respectively, where the horizontal coordinates denote each window of eRNAs and the vertical coordinates denote the number of eRNAs with the same histone modification feature on different windows.

**Figure S7.** For eRNA in PRAD tissue, the comparison of the strength of the same type of histone modification features on its different windows. (A), (B), (C), (D) and (E) show the H3K4me1, H3K4me3, H3K9me3, H3K27ac and H3K36me3 features of eRNAs, respectively, where the horizontal coordinates denote each window of eRNAs and the vertical coordinates denote the number of eRNAs with the same histone modification feature on different windows.

**Figure S8.** For eRNA in PAAD tissue, the comparison of the strength of the same type of histone modification features on its different windows. (A), (B), (C), (D) and (E) show the H3K4me1, H3K4me3, H3K9me3, H3K27ac and H3K36me3 features of eRNAs, respectively, where the horizontal coordinates denote each window of eRNAs and the vertical coordinates denote the number of eRNAs with the same histone modification feature on different windows.

**Table S28.** Optimal windows for extraction of each type of histone modification features in four normal tissues. S1, S2, etc. represent different samples, respectively. Mw denotes the middle window of eRNA. Aw denotes around the eRNA middle window, i.e. one window upstream and downstream of the middle window. Fw denotes the middle window far from the eRNA

| Tissue | Stomach | | | | | Lung | | | | | Liver | | | Pancreas | | |
| --- | --- | --- | --- | --- | --- | --- | --- | --- | --- | --- | --- | --- | --- | --- | --- | --- |
|  | S1 | S2 | S3 | S4 | S5 | S1 | S2 | S3 | S4 | S5 | S1 | S2 | S3 | S1 | S2 | S3 |
| H3K4me1 | Mw | Mw | Mw | Mw | Mw | Mw | Mw | Mw | Mw | Mw | Mw | Mw | Mw | Mw | Mw | Mw |
| H3K4me3 | Mw | Mw | Mw | Mw | Mw | Mw | Mw | Mw | Mw | Mw | Mw | Mw | Mw | Mw | Mw | Mw |
| H3K9me3 | Mw | Mw | Mw | Mw | Mw | Mw | Mw | Mw | Mw | Mw | Mw | Mw | Mw | Mw | Mw | Fw |
| H3K27ac | Mw | Mw | Mw | Mw | Mw | Mw | Mw | Mw | Mw | Mw | Mw | Mw | Mw | Mw | Mw | Mw |
| H3K36me3 | Aw | Mw | Aw | Mw | Mw | Fw | Mw | Aw | Aw | Mw | Mw | Aw | Aw | Fw | Fw | Mw |

**Table S29.** Proportions of regularly expressed eRNA(RE) and accidentally expressed eRNA(AE) in stomach tissue and the proportions of candidate RE and candidate AE in different samples.

| **Stomach (3943)** | | 34years | | 37years | | 51years | | 53years | | 54years | |
| --- | --- | --- | --- | --- | --- | --- | --- | --- | --- | --- | --- |
| **RE** | **AE** | RE | AE | RE | AE | RE | AE | RE | AE | RE | AE |
| **1084** | **2859** | 570 | 3373 | 1888 | 2055 | 1730 | 2213 | 667 | 3276 | 987 | 2956 |
| **27.49%** | **72.51%** | 14.46% | 85.54% | 47.88% | 52.12% | 43.88% | 56.12% | 16.92% | 83.08% | 25.03% | 74.97% |

**Table S30.** Proportions of regularly expressed eRNA(RE) and accidentally expressed eRNA(AE) in lung tissue and the proportions of candidate RE and candidate AE in different samples.

| **Lung (6481)** | | 3years | | 37years | | 51years | | 54years | | 59years | |
| --- | --- | --- | --- | --- | --- | --- | --- | --- | --- | --- | --- |
| **RE** | **AE** | RE | AE | RE | AE | RE | AE | RE | AE | RE | AE |
| **2128** | **4353** | 1083 | 5398 | 2182 | 4299 | 2690 | 3791 | 2122 | 4359 | 2257 | 4224 |
| **32.83%** | **67.17%** | 16.71% | 83.29% | 33.67% | 66.33% | 41.51% | 58.49% | 32.74% | 67.26% | 34.82% | 65.18% |

**Table S31.** Proportions of regularly expressed eRNA(RE) and accidentally expressed eRNA(AE) in liver tissue and the proportions of candidate RE and candidate AE in different samples.

| **Liver (3171)** | | 16years | | 25years | | 31years | |
| --- | --- | --- | --- | --- | --- | --- | --- |
| **RE** | **AE** | RE | AE | RE | AE | RE | AE |
| **747** | **2424** | 850 | 2321 | 930 | 2241 | 552 | 2619 |
| **23.56%** | **76.44%** | 26.81% | 73.19% | 29.33% | 70.67% | 17.41% | 82.59% |

**Table S32.** Proportions of regularly expressed eRNA(RE) and accidentally expressed eRNA(AE) in pancreas tissue and the proportions of candidate RE and candidate AE in different samples.

| **Pancreas (3368)** | | 30years | | 34years | | 59years | |
| --- | --- | --- | --- | --- | --- | --- | --- |
| **RE** | **AE** | RE | AE | RE | AE | RE | AE |
| **480** | **2888** | 607 | 2761 | 707 | 2661 | 327 | 3041 |
| **14.25%** | **85.75%** | 18.02% | 81.98% | 20.99% | 79.01% | 9.71% | 90.29% |

**Table S33.** Proportion of regularly expressed eRNA(RE) and accidentally expressed eRNA(AE) in LIHC, LUAD, PRAD and PAAD tissues.

| **LIHC (442)** | | | **LUAD (1178)** | | | **PRAD (964)** | | | **PAAD (835)** | | |
| --- | --- | --- | --- | --- | --- | --- | --- | --- | --- | --- | --- |
| **RE** | **AE** | | **RE** | **AE** | | **RE** | **AE** | | **RE** | **AE** | |
| **102** | **340** | | **250** | **928** | | **201** | **763** | | **80** | | **755** |
| **23.08%** | | **76.92%** | **21.22%** | | **78.78%** | **20.85%** | | **79.15%** | **9.58%** | | **90.42%** |

**Figure S9.** (A) shows the comparison of eRNA identification models on the independent test set of four normal tissues, and (B) shows the comparison of eRNA identification models on the independent test set of four cancer tissues.

**Table S34.** Comparison of models on the stomach tissue test set

| Method | ACC (%) | Sn (%) | Sp (%) | MCC | AUC |
| --- | --- | --- | --- | --- | --- |
| SeqPose | 61.09 | 44.99 | 77.19 | 0.1929 | 0.6612 |
| iEnhancer-RD | 63.88 | 60.20 | 67.55 | 0.2783 | 0.6966 |
| FRL | 66.22 | 66.03 | 66.41 | 0.3245 | 0.7049 |
| LSTMAtt | 63.94 | 58.81 | 69.07 | 0.2803 | 0.6883 |
| DeepITEH | **86.25** | **74.40** | **98.10** | **0.6518** | **0.9015** |

**Table S35.** Comparison of models on the lung tissue test set

| Method | ACC (%) | Sn (%) | Sp (%) | MCC | AUC |
| --- | --- | --- | --- | --- | --- |
| SeqPose | 56.37 | 44.44 | 68.29 | 0.1144 | 0.5880 |
| iEnhancer-RD | 57.18 | 53.32 | 61.03 | 0.1439 | 0.6196 |
| FRL | 58.18 | **61.27** | 55.09 | 0.1639 | 0.6190 |
| LSTMAtt | 58.80 | 55.79 | 61.81 | 0.1762 | 0.6224 |
| DeepITEH | **78.59** | 58.64 | **98.53** | **0.4834** | **0.8412** |

**Table S36.** Comparison of models on the liver tissue test set

| Method | ACC (%) | Sn (%) | Sp (%) | MCC | AUC |
| --- | --- | --- | --- | --- | --- |
| SeqPose | 58.75 | 41.64 | 75.87 | 0.1511 | 0.6372 |
| iEnhancer-RD | 60.80 | 50.95 | 70.66 | 0.2204 | 0.6636 |
| FRL | 62.78 | **61.20** | 64.35 | 0.2556 | 0.6811 |
| LSTMAtt | 65.85 | 53.47 | 78.23 | 0.3272 | 0.7500 |
| DeepITEH | **70.74** | 47.79 | **93.69** | **0.3434** | **0.7663** |

**Table S37.** Comparison of models on the pancreas tissue test set

| Method | ACC (%) | Sn (%) | Sp (%) | MCC | AUC |
| --- | --- | --- | --- | --- | --- |
| SeqPose | 55.12 | 42.28 | 67.95 | 0.0913 | 0.5557 |
| iEnhancer-RD | 58.53 | **70.62** | 46.44 | 0.1758 | 0.6269 |
| FRL | 58.98 | 63.65 | 54.30 | 0.1803 | 0.6318 |
| LSTMAtt | 59.42 | 57.42 | 61.42 | 0.1886 | 0.6286 |
| DeepITEH | **65.43** | 38.43 | **92.43** | **0.2487** | **0.7271** |

**Table S38.** Comparison of models on the LIHC tissue test set

| Method | ACC (%) | Sn (%) | Sp (%) | MCC | AUC |
| --- | --- | --- | --- | --- | --- |
| SeqPose | 55.11 | 35.23 | 75.00 | 0.0865 | 0.5580 |
| iEnhancer-RD | 61.36 | 50.00 | 72.73 | 0.2334 | 0.6448 |
| FRL | 61.93 | **62.50** | 61.36 | 0.2387 | 0.6513 |
| LSTMAtt | 58.52 | 52.27 | 64.77 | 0.1718 | 0.6460 |
| DeepITEH | **70.45** | 45.45 | **95.45** | **0.3340** | **0.7450** |

**Table S39.** Comparison of models on the LUAD tissue test set

| Method | ACC (%) | Sn (%) | Sp (%) | MCC | AUC |
| --- | --- | --- | --- | --- | --- |
| SeqPose | 59.00 | 32.50 | 85.50 | 0.1455 | 0.6602 |
| iEnhancer-RD | 65.25 | **64.00** | 66.50 | 0.3051 | 0.7247 |
| FRL | 65.75 | 56.00 | 75.50 | 0.3212 | 0.7243 |
| LSTMAtt | 66.50 | 51.00 | 82.00 | 0.3471 | 0.7230 |
| DeepITEH | **71.00** | 53.50 | **88.50** | **0.3615** | **0.8214** |

**Table S40.** Comparison of models on the PRAD tissue test set

| Method | ACC (%) | Sn (%) | Sp (%) | MCC | AUC |
| --- | --- | --- | --- | --- | --- |
| SeqPose | 58.81 | 47.15 | 70.47 | 0.1586 | 0.5988 |
| iEnhancer-RD | 55.44 | 45.08 | 65.80 | 0.1112 | 0.6212 |
| FRL | 57.51 | **62.18** | 52.85 | 0.1509 | 0.6327 |
| LSTMAtt | 58.55 | 55.44 | 61.66 | 0.1713 | 0.6238 |
| DeepITEH | **68.65** | 39.90 | **97.41** | **0.2972** | **0.7264** |

**Table S41.** Comparison of models on the PAAD tissue test set

| Method | ACC (%) | Sn (%) | Sp (%) | MCC | AUC |
| --- | --- | --- | --- | --- | --- |
| SeqPose | 57.19 | 44.91 | 69.46 | 0.1288 | 0.6110 |
| iEnhancer-RD | 59.88 | 52.10 | 67.66 | 0.2000 | 0.6628 |
| FRL | 60.18 | **67.07** | 53.29 | 0.2056 | 0.6685 |
| LSTMAtt | **63.47** | 59.28 | 67.66 | **0.2704** | 0.6783 |
| DeepITEH | 60.78 | 22.16 | **99.40** | 0.1619 | **0.7061** |
