# Supplementary figures and images for: DeepITEH: a deep learning framework for identifying tissue-specific eRNAs from the human genome
Source: Bioinformatics. 2023 Jun 9;39(6):btad375. doi: 10.1093/bioinformatics/btad375 (PMC10281860; doi:10.1093/bioinformatics/btad375)

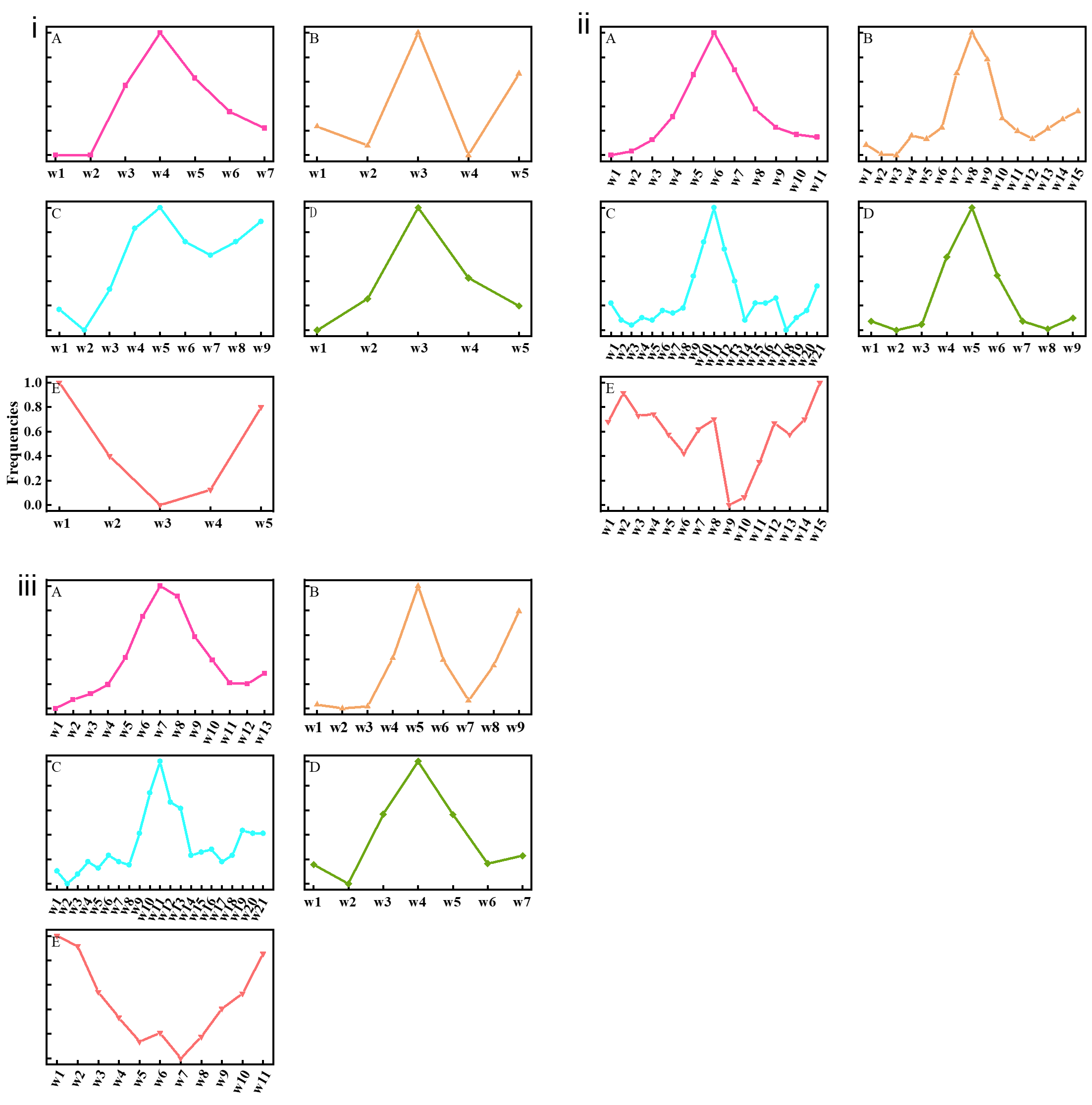

Supplement: btad375_Supplementary_Data [file btad375_supplementary_data.zip › Figure S3.tif]

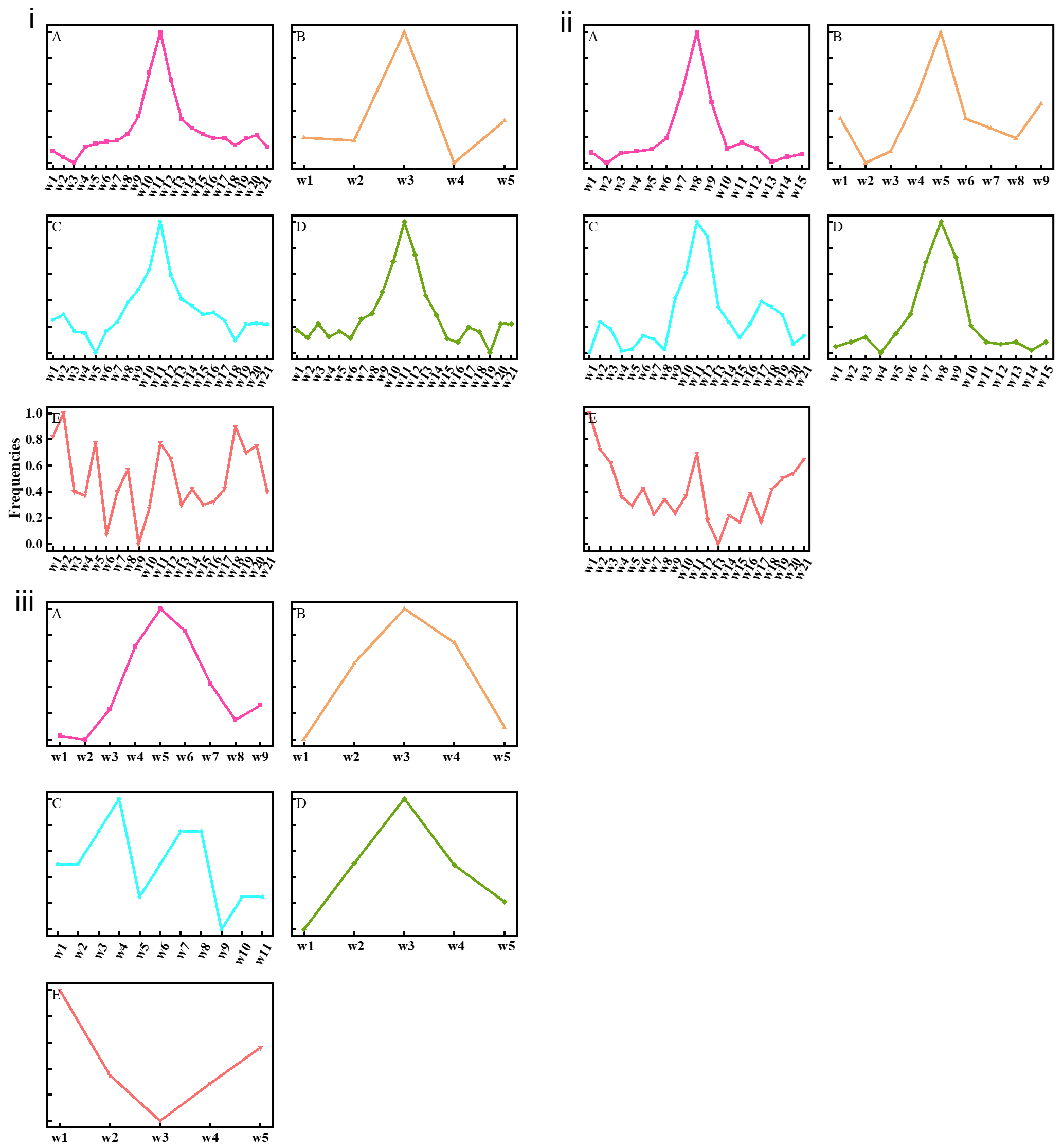

Supplement: btad375_Supplementary_Data [file btad375_supplementary_data.zip › Figure S4.tif]

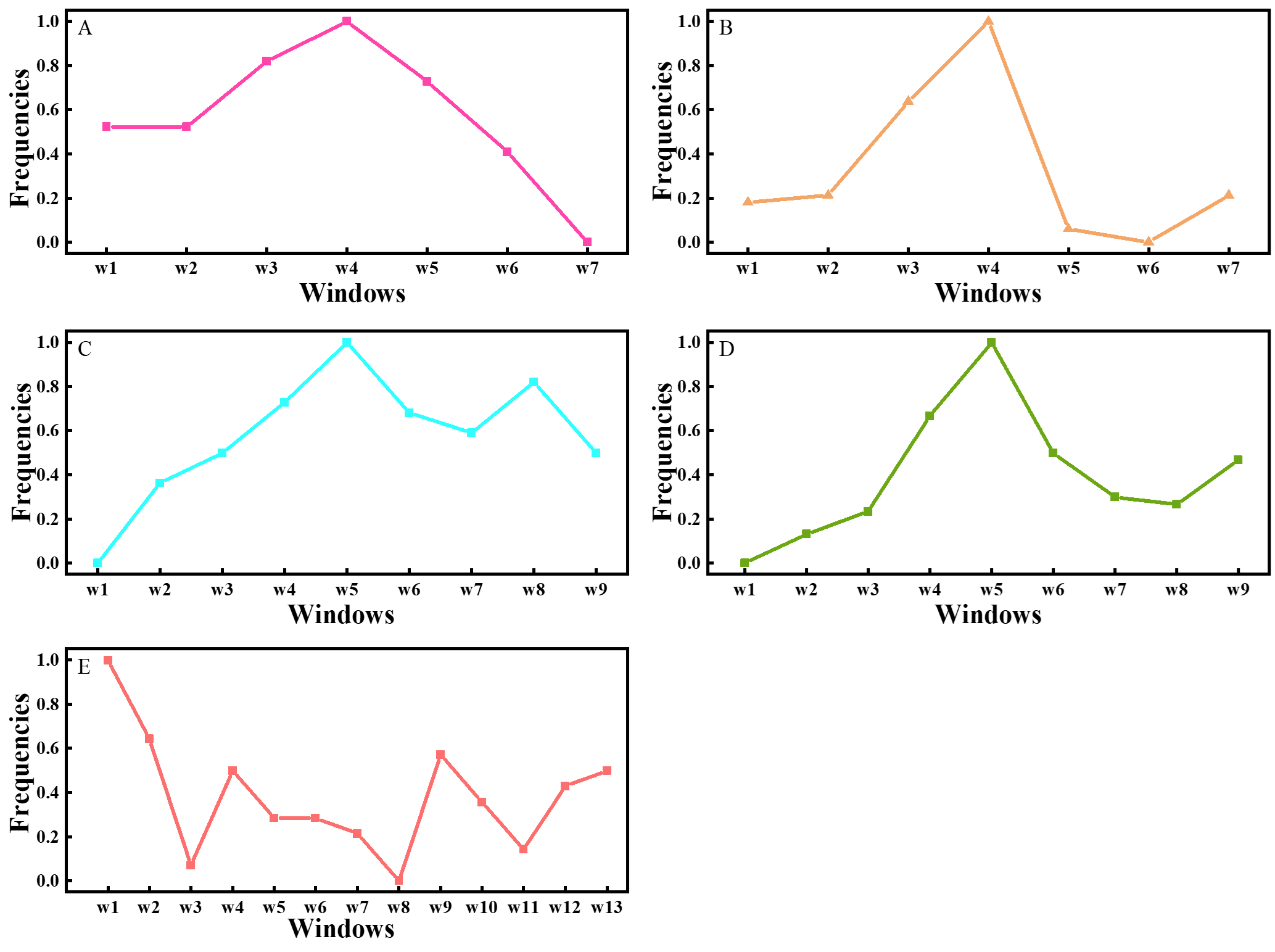

Supplement: btad375_Supplementary_Data [file btad375_supplementary_data.zip › Figure S5.tif]

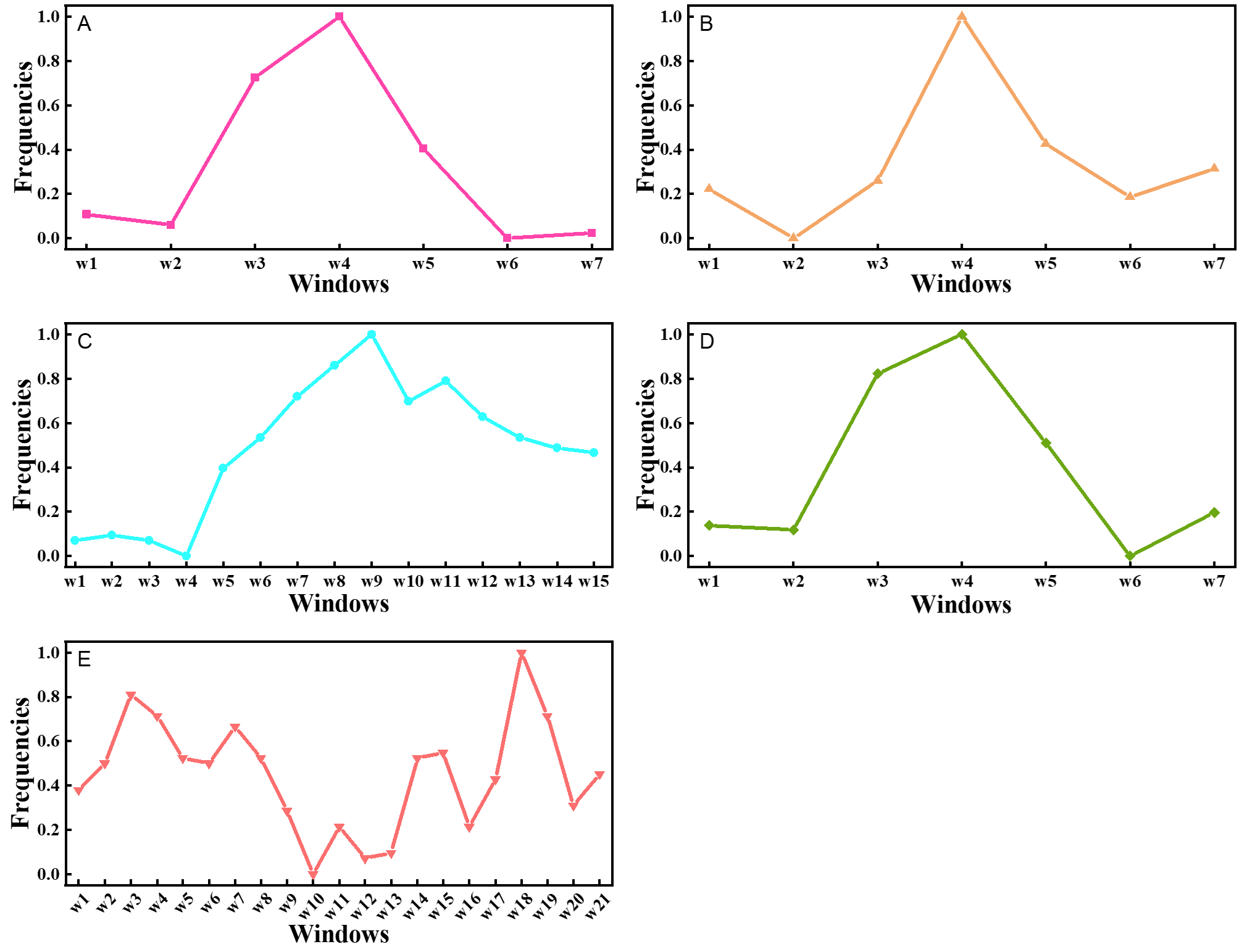

Supplement: btad375_Supplementary_Data [file btad375_supplementary_data.zip › Figure S6.tif]

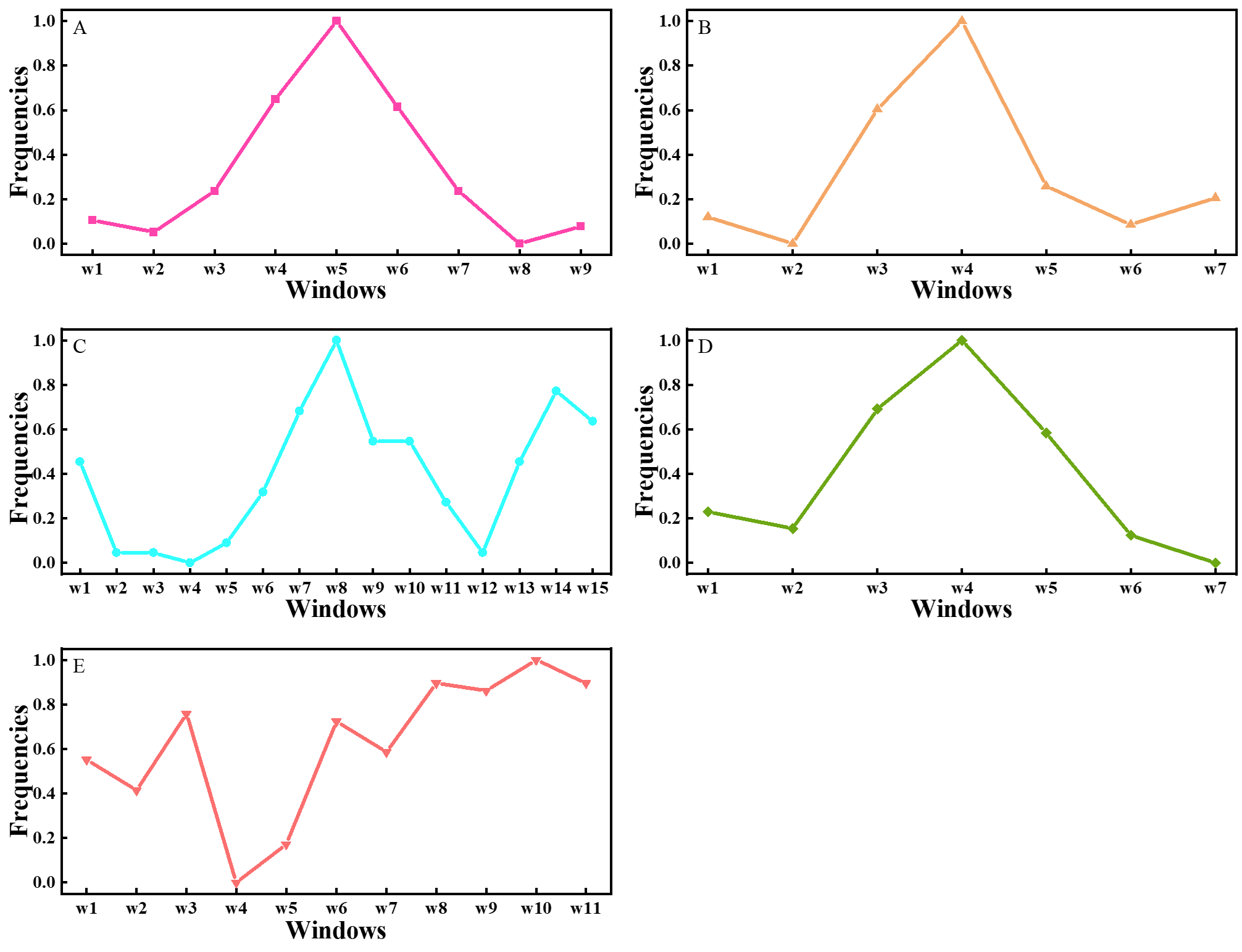

Supplement: btad375_Supplementary_Data [file btad375_supplementary_data.zip › Figure S7.tif]

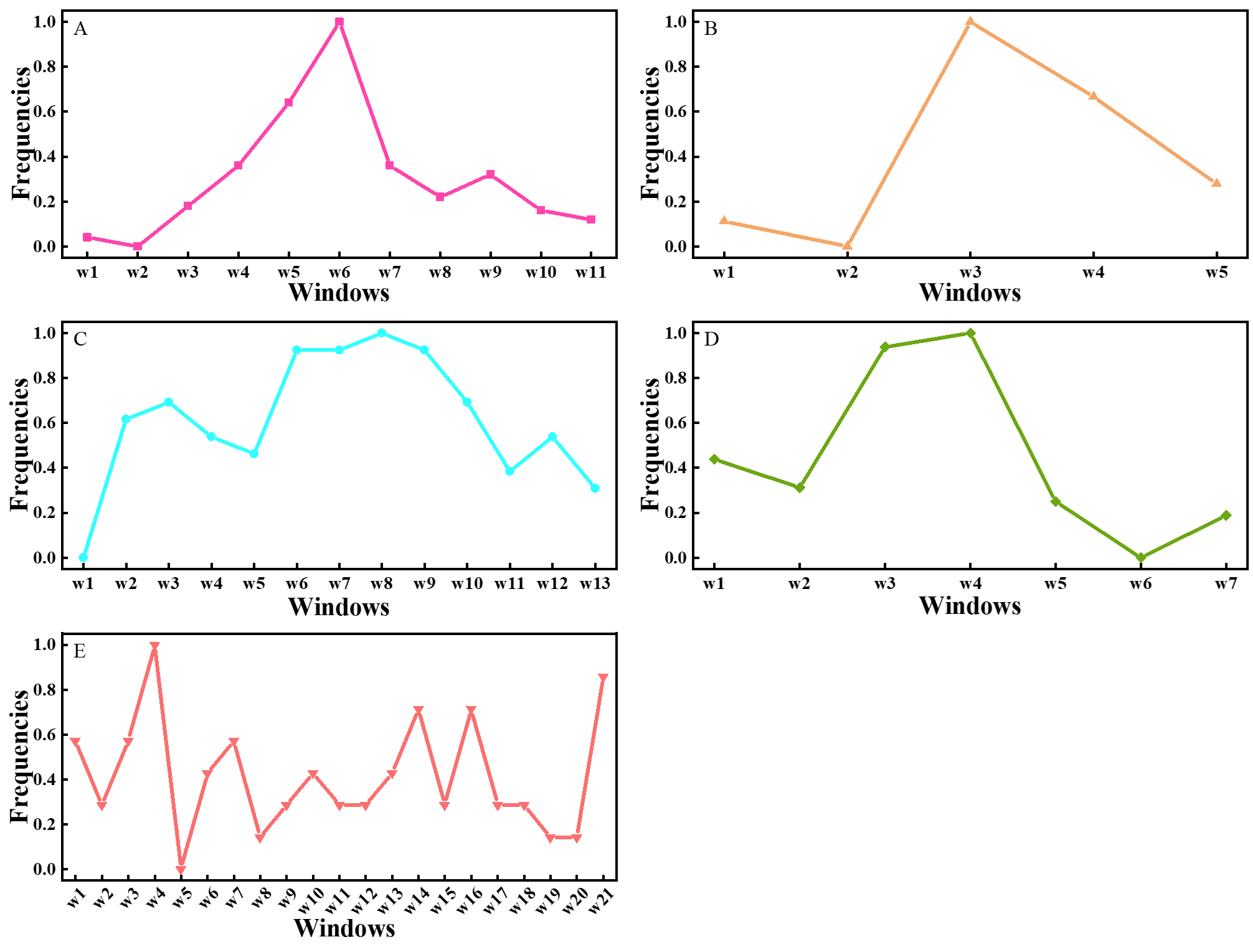

Supplement: btad375_Supplementary_Data [file btad375_supplementary_data.zip › Figure S8.tif]

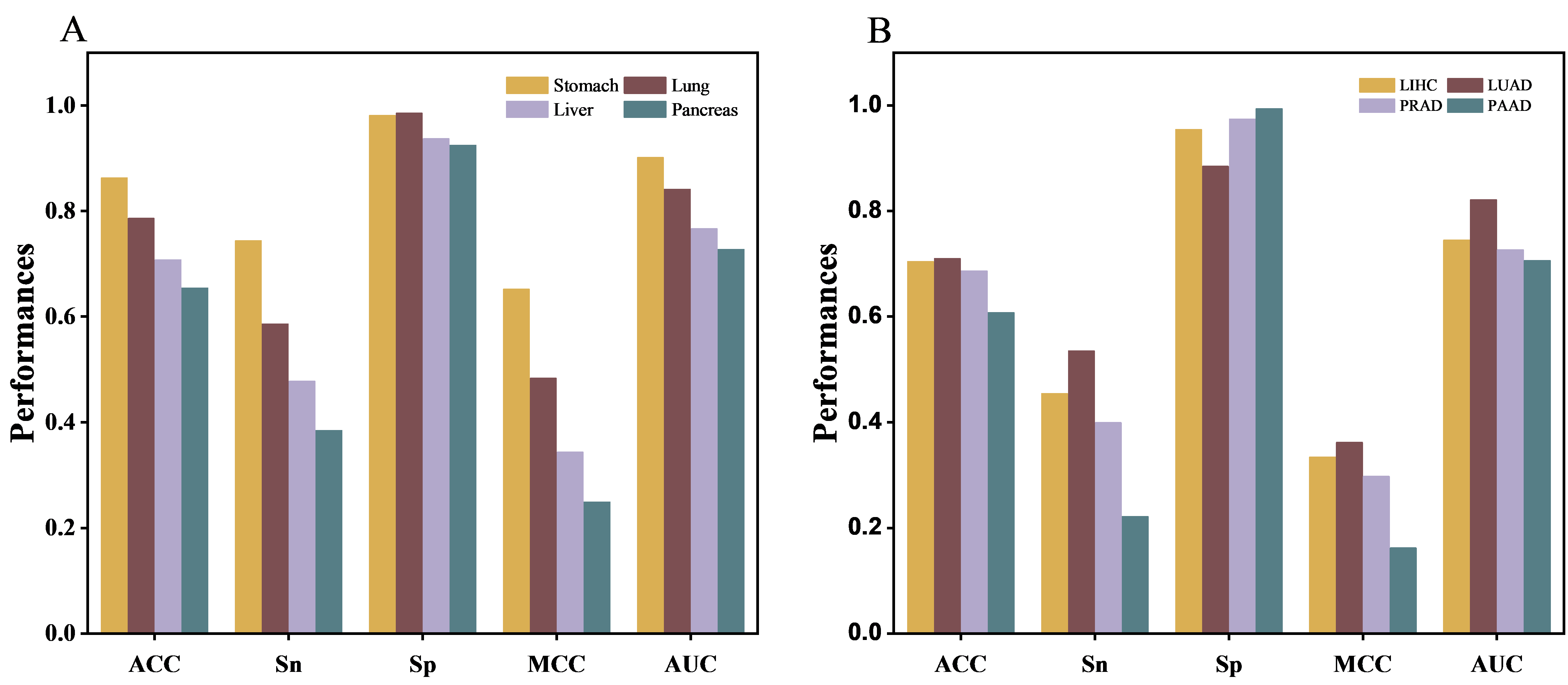

Supplement: btad375_Supplementary_Data [file btad375_supplementary_data.zip › Figure S9.tif]

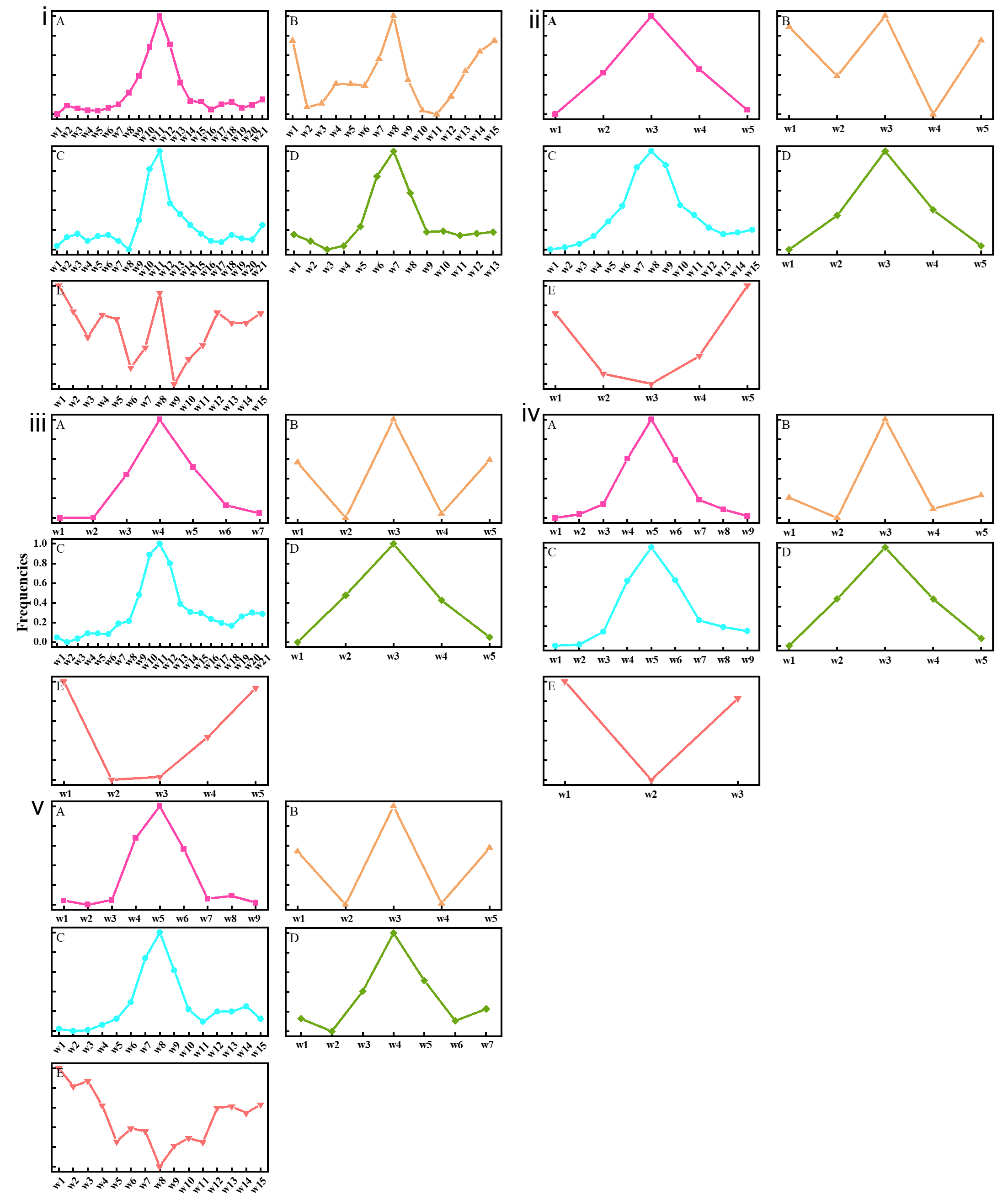

Supplement: btad375_Supplementary_Data [file btad375_supplementary_data.zip › Figure S1.tif]

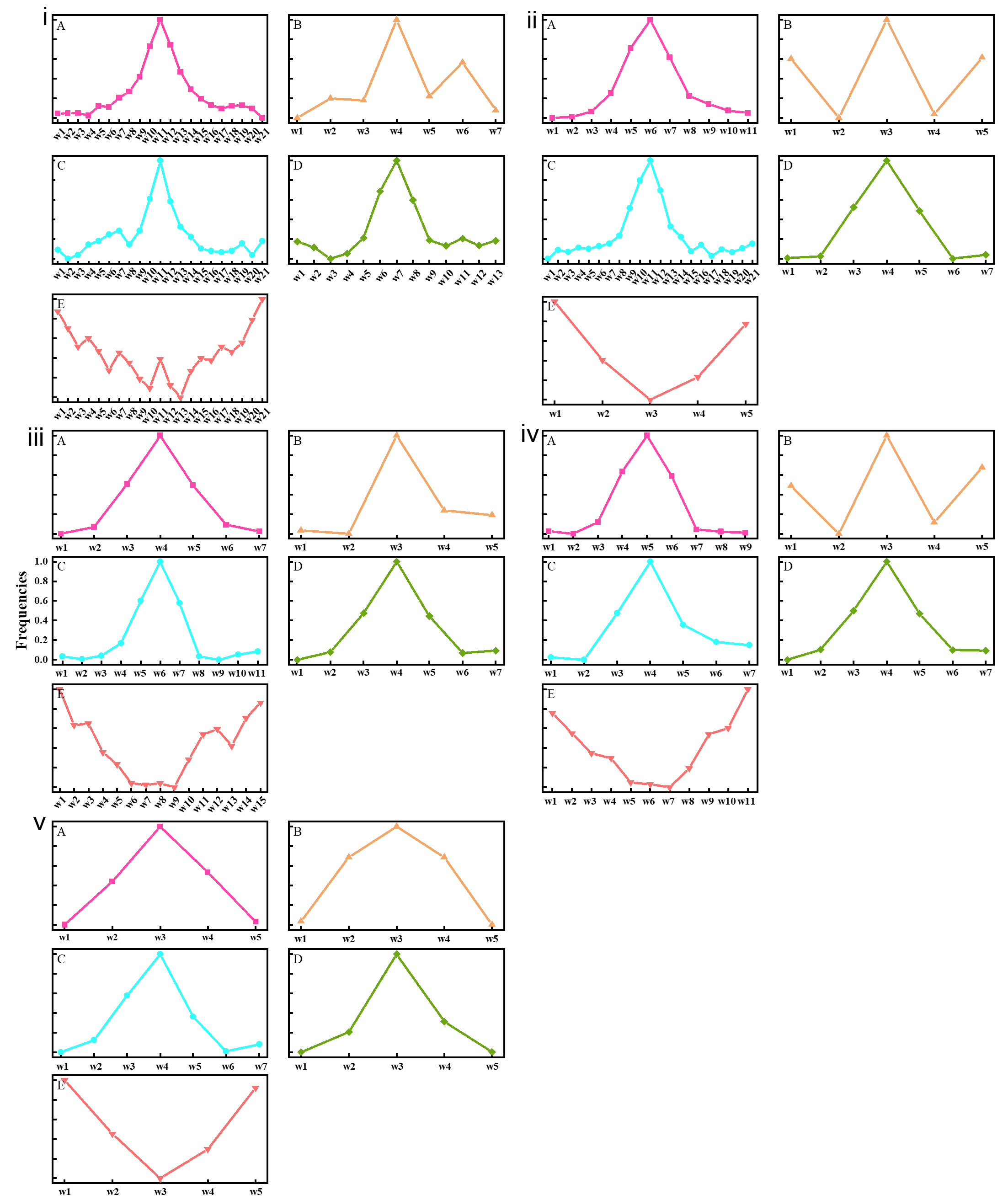

Supplement: btad375_Supplementary_Data [file btad375_supplementary_data.zip › Figure S2.tif]
